# Neuromuscular blockade and their monitoring in the intensive care unit: a multicenter observational prospective study

**DOI:** 10.1186/s13613-025-01591-4

**Published:** 2025-10-22

**Authors:** Bertrand Hermann, Guillaume Decormeille, Tiphanie Gobé, Nathanaël Mangeard, Adel Maamar, Saria Sayadi, Bénédicte Pernod, Nadine Robquin, Jean-Pierre Ponthus, Sophie Le Potier, Pierre Bouju, Angélique Balabanian, Antoine Frouin, Sébastien Moschietto, Gwenaelle Jacq, Emeline Villemont, Clémence Houbé, Anaïs Queyreau, Célina Morand, Florence Boissier, Jean-Baptiste Lascarrou, Sabine Valera, Sami Hraiech, Laure Clouet, Gaël Piton, Cindérella Noël, Anne Joosten, Cécilia Tabra Osorio, Adrien Constan, Jérôme Cecchini, Gwennaelle Mercier, Arnaud Bruyneel, Chloé Villamaux, François Pousset, Nicholas Heming, Laurent Poiroux, Jean-François Llitjos, Saber Davide Barbar

**Affiliations:** 1 Service de Médecine intensive-Réanimation, Hôpital Européen Georges Pompidou, Assistance Publique–Hôpitaux de Paris (AP-HP), 20 rue Leblanc, 75015 Paris, France; 2 INSERM 1266, Institute of Psychiatry and Neurosciences of Paris (IPNP), INSERM UMR 1266, Université Paris Cité, F-75006 Paris, France; 3 Service de Réanimation Médicale, Centre Hospitalier Universitaire de Toulouse, Toulouse, France; 4 Service de Réanimation Médicale, CHU Pontchaillou, Rennes, France; 5 Service de Médecine intensive-Réanimation, Hôpital Ambroise Paré, Assistance Publique–Hôpitaux de Paris (AP-HP), Paris, France; 6 Service de Médecine intensive-Réanimation, Hôpital Lucie et Raymond Aubrac, Villeneuve-Saint-Georges, France; 7 Service de Réanimation Polyvalente, CH de Lorient, Lorient, France; 8 Service de Réanimation Médico-Chirurgicale, Hôpital René Dubos, Pontoise, France; 9 Service de Réanimation Polyvalente, CH d’Avignon, Avignon, France; 10 Service de Médecine intensive-Réanimation, CH André Mignot, Le Chesnay, France; 11 Division of Anesthesia, Critical Care, Pain and Emergency Medicine, Nîmes University Hospital, University of Montpellier, Nîmes, France; 12 Service de Réanimation Polyvalente Adulte, Hôpital Necker, Assistance Publique–Hôpitaux de Paris (AP-HP), Paris, France; 13 Service de Médecine intensive-Réanimation, CHU Poitiers, France; 14 Service de Médecine intensive-Réanimation, CHU Nantes, France; 15 Service de Médecine intensive-Réanimation, Assistance Publique–Hôpitaux de Marseille, Marseille, France; 16 Service de Médecine intensive-Réanimation, CHU Besançon, France; 17 Unité de Soins Intensifs, Hôpital Civil Marie Curie, Lodelinsart, Belgique; 18 Service de Médecine intensive–Réanimation, Centre hospitalier Intercommunal de Créteil, Créteil, France; 19 Service de Soins Intensifs, Hôpital Erasme, HUB, Bruxelles, Belgique; 20 Health Economics, Hospital Management and Nursing Research Department, School of Public Health, Université Libre de Bruxelles, Bruxelles, Belgium; 21 Service de Réanimation Polyvalente, CH Mayotte, Mamoudzou, France; 22 Service de Médecine intensive-Réanimation, Hôpital Raymond Poincaré, Assistance Publique–Hôpitaux de Paris (AP-HP), Garches, France; 23 Service de Médecine intensive–Réanimation, CHU d’Angers, Angers, France; 24 Laboratoire BioMérieux, Craponne, France; 25 UR‑UM103 IMAGINE, University of Montpellier, Nimes University Hospital, Nîmes, France

**Keywords:** Neuromuscular blockade, Critical care, Mechanical ventilation, Acute respiratory distress syndrome

## Abstract

**Background:**

Neuromuscular blocking agents may improve outcomes in specific conditions, including the early phase of acute respiratory distress syndrome. However, neuromuscular blocking agents are associated with side effects and uncertainty persists regarding their optimal dosing and efficacy. Our objective was to describe the use of neuromuscular blocking agents in a real-world setting.

**Methods:**

We conducted a multicenter, prospective observational study, including adult patients who underwent invasive mechanical ventilation and received a continuous infusion of neuromuscular blocking agents. Patients were recruited across 19 intensive care units in France and Belgium.

**Results:**

From November 16, 2019, to February 19, 2020, a total of 2248 patients were hospitalized and mechanically ventilated in 19 participating ICUs. Of these, 270 (12%) patients received at least one dose of neuromuscular blocking agents, and 232 (10.3%) received a continuous infusion. The main indications for neuromuscular blocking agents use were acute respiratory distress syndrome (61%), prevention of shivering during therapeutic hypothermia (16%) and patient-ventilator asynchrony (12%). Infusion was initiated in median at 0 [0–2] days after ICU admission, with a median duration of 38 [22–71] hours. Cisatracurium was the preferred agent (74%). Neuromuscular blocking agents monitoring by train-of-four was employed in 48% of patients. Intensive care unit-acquired weakness was diagnosed in 25% of patients, pressure ulcers in 14% and ventilator-associated pneumonia in 26%. The median lengths of mechanical ventilation and ICU stay were 9 [4–16] and 13 [6–22] days, and ICU mortality was 41%. In multivariable analyses, a duration of neuromuscular blocking agents infusion exceeding 48 hours was associated with a lower cumulative incidence of weaning success (SHR 0.83 [0.76, 0.91], p < 0.001) and higher incidences of ventilator-associated pneumonia, while neuromuscular blocking agents monitoring was associated with both increased intensive care unit-acquired weakness (OR 2.90 [1.2, 7.01], p = 0.018) and reduced ICU mortality (HR 0.55 [95%CI 0.32, 0.95], p = 0.032).

**Conclusion:**

In our study, the prevalence of continuous neuromuscular blocking agents infusion among mechanically ventilated patients in the intensive care unit was 10.3%. While acute respiratory distress syndrome was the main indication, over one-third of patients received neuromuscular blocking agents for other reasons. A duration of neuromuscular blocking agents infusion exceeding 48 hours was associated with longer mechanical ventilation and increased complications. The role of neuromuscular blocking agents monitoring remains unclear.

*Trial registration* ClinicalTrials.gov: NCT04028362 Registered on 18 July 2019, https://clinicaltrials.gov/study/NCT04028362.

The study was conducted by the French Intensive Care Society/Société de Réanimation de Langue Française Trial Group.

**Supplementary Information:**

The online version contains supplementary material available at 10.1186/s13613-025-01591-4.

## Introduction

Management of patients in the intensive care unit (ICU) frequently involves sedation and analgesia to promote patient comfort and safety during invasive procedures. In addition to sedation and analgesia, neuromuscular blocking agents (NMBA) may also be required to facilitate invasive procedures, such as endotracheal tube placement or surgery. Beyond these short-term indications, continuous NMBA infusion may be used in patients under invasive mechanical ventilation in the ICU for specific therapeutic purposes such as preventing shivering during therapeutic hypothermia [1] or avoiding patient-ventilator (P/V) asynchrony. In acute respiratory distress syndrome (ARDS), continuous NMBA might help to reduce the work of breathing, improve oxygenation, prevent ventilator-induced lung injury, and facilitate prone positioning. [2–4]. NMBA infusion might also be considered in acute severe asthma, intracranial hypertension notably in severe traumatic brain injury [5, 6], or abdominal compartment syndrome [7], although with low levels of evidence.

NBMA use has been reported in 13% [8] to 20% [9] of mechanically ventilated ICU patients and in up to 38% of patients with severe ARDS [10]. However, since the advent of daily sedation interruption protocols or protocolized goal-directed sedation [11] and the increased recognition of the potential side effects of NMBA, including ICU-acquired weakness, although ICU-acquired weakness was not associated with short-term NMBA use (≤48h) in the ROSE trial [3], the use of NMBA has likely declined in recent years. Nevertheless, there is little recent data on the epidemiology of NMBA use in the ICU.

Similarly to sedation and analgesia, protocolized NMBA infusion strategies have been proposed to monitor the depth of paralysis and guide recovery. Tools to guide NMBA infusion include qualitative clinical assessment [12], semi-quantitative monitoring scales such as the Bedside Shivering Assessment Scale (BSAS) [13]and quantitative monitoring techniques such as the assessment of muscular contraction in response to peripheral nerve stimulation by a train-of-four (TOF) electrical impulses or other devices such as accelerometry or electromyography. However, contrary to the operating room, where the benefit of NMBA monitoring is well-established, studies in the ICU have yielded mixed results. For instance, Strange *et al.* [14]and Bauman *et al*. [15] found no benefit to a TOF-guided NMBA infusion strategy. In contrast, Hraiech *et al.* recently observed that a nurse-driven NMBA protocol based on TOF significantly reduced NMBA exposure during ARDS management [16]. Although guidelines recommend NMBA monitoring [17], the overall evidence regarding its benefits, as well as the optimal devices and objectives, remains low.

The primary objective of the study was to report the epidemiology of continuous NMBA use in patients undergoing invasive mechanical ventilation in the ICU. Secondary objectives were to describe protocols and practices for NMBA monitoring and to identify factors associated with ICU outcomes.

## Material and methods

### Study design

A multicenter, prospective, observational study, conducted across 19 centers in France and Belgium. The study was initiated and supported by the Société de Réanimation de Langue Française (SRLF, French Intensive Care Society). No other third-party nor any laboratory had any role in either the conduct or the funding of the study.

### Study population

All adult patients admitted to the ICU, undergoing invasive mechanical ventilation, and receiving at least a single dose and/or a continuous infusion of NMBA with an expected ICU stay duration of at least 48h were included. Patients receiving NMBA exclusively for rapid sequence induction during intubation and patients receiving NMBA outside the ICU (e.g., in the operating room) were excluded. Additional exclusion criteria included patients previously enrolled in the study, patients under legal protection, and pregnant or breastfeeding women.

### Data collection

Data were prospectively collected. At admission: patient demographic characteristics, etiology of admission and severity of illness using the Simplified Acute Physiology Score II (SAPS II); during ICU stay: use of organ support (i.e. invasive mechanical ventilation, renal replacement therapy, and vasopressors), prone position in patients receiving NMBA for ARDS; NMBA-specific data, i.e. molecule used, mode of administration (single bolus vs. continuous intravenous infusion via syringe pump with or without a preceding bolus infusion), indication (categorized a priori), bolus dose, number of infusion episodes (a new episode was defined as an NMBA-free period >24 hours), duration (prolonged NMBA infusion was defined as continuous infusion exceeding 48 hours), hourly and cumulative dose for continuous infusion as well as frequency and reasons for dose adjustments, NMBA TOF monitoring (conducted through peripheral nerve stimulation performed either at the ulnar nerve site, adductor pollicis muscle, or at the facial nerve site, orbicularis oculi muscle, with TOF values and monitoring frequency reported by the nurse in charge); center-level data i.e. presence of NMBA monitoring protocols, characteristics of the protocols; and at the end of ICU Stay, vital status, duration of invasive mechanical ventilation, ICU length of stay, outcomes potentially related to NMBA i.e. ICU-acquired weakness, pressure ulcers, ventilator-associated pneumonia as reported by the physician in charge (see definitions Supplementary material).

### Primary and secondary outcomes

The primary outcome was the period prevalence of NMBA use in continuous infusion among invasive mechanical ventilation patients in the ICU, defined as the proportion of patients receiving continuous NMBA infusion in the ICU out of all patients admitted to the ICU and undergoing invasive mechanical ventilation over the study period.

Secondary outcomes were the description of NMBA monitoring protocols and practices, the description of continuous NMBA infusion modalities (main indication using a priori defined categories, duration, doses, monitoring and protocols) and the potentially NMBA-related side effects (pressure ulcers, ICU-acquired weakness, ventilator-associated pneumonia and length of invasive mechanical ventilation). Finally, we also investigated the factors associated with ICU mortality and with these potentially NMBA-related side effects.

### Statistical analysis

All analyses were performed using R version 4.5.0 (2025–04–11). Continuous variables are presented as median (interquartile range, IQR) and categorical variables as absolute frequencies and proportions. Group differences were assessed using Mann-Whitney U tests or Kruskal-Wallis tests for continuous variables and χ^2^ tests or Fisher’s exact tests for categorical variables, as appropriate. All statistical tests were two-sided with a significance level (type I error) set at 0.05. To account for multiple comparisons, p-values were adjusted using the false discovery rate (FDR) approach, whenever appropriate.

To identify potential associations between NMBA and ICU outcomes, we performed univariable and multivariable analyses for each outcome in the population receiving continuous NMBA infusion. All these analyses were performed after missing data imputations through Multiple Imputation by Chained Equations (MICE) considering data were missing at random. Data were imputed 5 times with predictive mean matching for continuous variables and binary and polytomous logistic regression for categorical variables. Univariable analyses included all demographic, comorbidities, severity and reason for ICU admission (dichotomized as respiratory failure, cardiac arrest or other), NMBA-related variables (indication for NMBA treatment, dichotomized as ARDS or non-ARDS, duration, median dose and whether or not NMBA was monitored), other variables at day one of NMBA (analgesia and sedative regimen, maximal PEEP and plateau pressure levels and minimal PaO_2_/FiO_2_) and organ support during the stay. These analyses were also performed in the subgroup of patients receiving NMBA for ARDS, with the addition of prone positioning during the stay as variable. All variables with unadjusted p<0.2 in univariable analyses were included in the multivariable analyses. ICU survival was assessed with a Cox proportional hazards regression analysis. Weaning success was assessed using Fine and Gray competing risk regression sub-distribution hazard model with death as the competing risk. The other binary outcomes (pressure ulcers, ventilator-associated pneumonia and ICU-acquired weakness) were assessed using logistic regression models. All analyses were clustered on centers, with estimates from each imputed dataset pooled using the Rubin’s rules and robust confidence intervals estimation.

### Ethics

The study was approved by the *Comité de Protection des Personnes Sud-Ouest et Outre-Mer 2* n°ID-RCB 2019-A01378-49 and was prospectively registered in ClinicalTrials.gov (NCT04028362). Informed consent was obtained from patients’ relatives. Reporting adhered to the Strengthening the Reporting of Observational Studies in Epidemiology (STROBE) guidelines.

## Results

### Prevalence of NMBA use

**Fig. 1.**
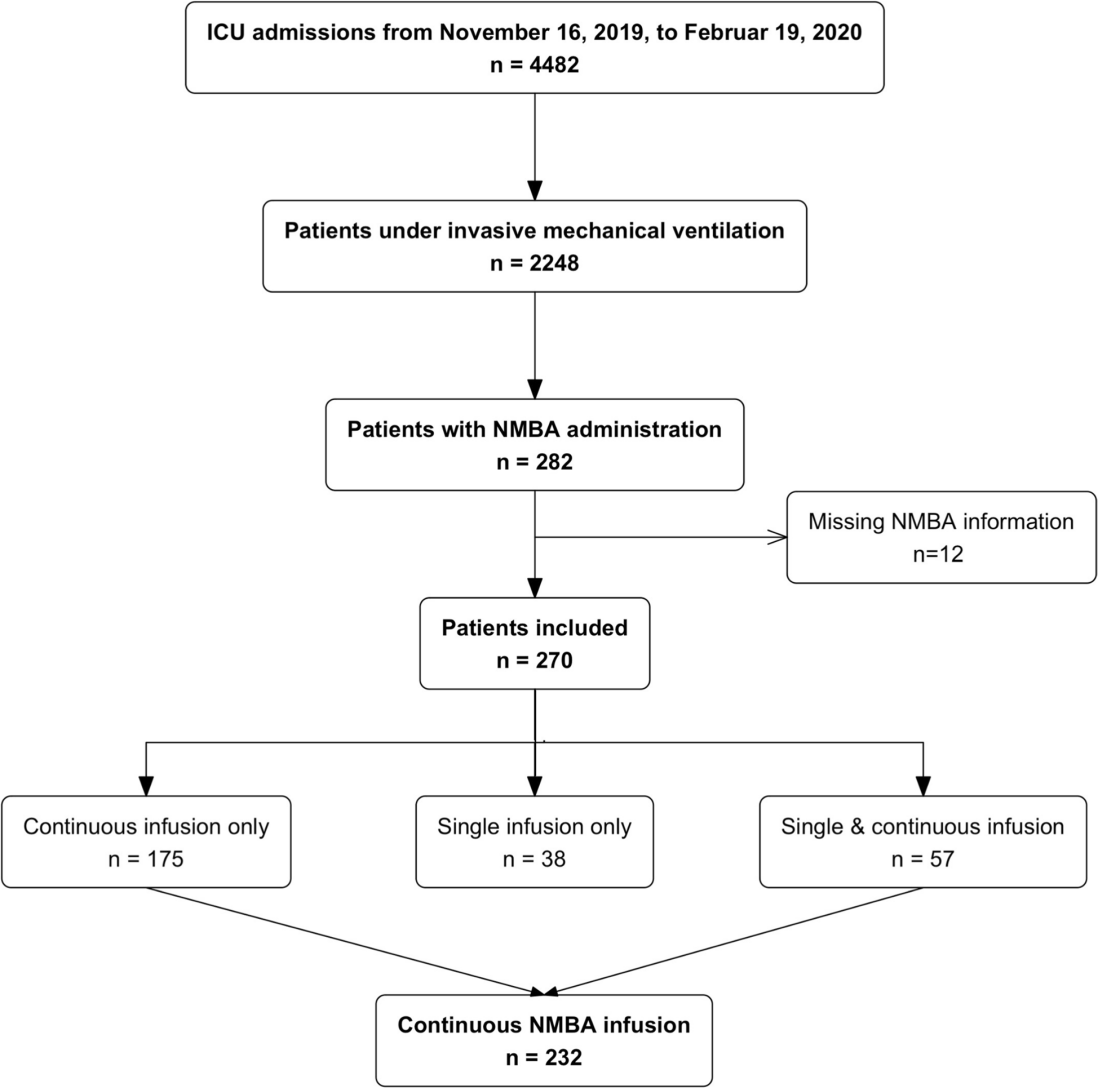
Flowchart

From November 16, 2019, to February 19, 2020, a total of 4482 patients were hospitalized in the ICUs of the 19 participating centers. Among these, 2248 patients underwent invasive mechanical ventilation and 282 patients received NMBA. Twelve patients with missing information about NMBA administration were excluded, of which 38 (14.1%) received a single bolus infusion, 175 (64.8%) a continuous infusion and 57 (21.1%) received both a single infusion and a continuous infusion (see flowchart, Fig. 1).

The overall period prevalence of NMBA use among mechanically ventilated patients during the study period was 12.0% (270/2248). Specifically, the prevalence was 10.3% (232/2248) for continuous infusions and 4.2% (95/2248) for single bolus infusions.

NMBA bolus infusions are described in Supplementary tables 1 and 2. All subsequent analyses and results only concern the primary population of interest of patients receiving continuous NMBA infusions.

### NMBA continuous infusion

A total of 232 patients received continuous NMBA infusion, comprising 264 episodes of NMBA administration (where episodes are defined as periods of continuous NMBA infusions separated by at least 24 h) with a median number of 1 [1-1] episode per patient. The majority of patients were male (165, 71%), with a median age of 63 [52–71] years and a median SAPSII of 59 [42–76] (See Table 1).

**Table 1:** Characteristics of the population receiving continuous NMBA infusions

Variable	Missing (N (%))	Overall
		N = 232
Demographic and ICU admission		
Age (years)	2 (0.9%)	63 [52-71]
Female sex	0 (0%)	67 (29)
BMI (kg/m^2^)	10 (4.3%)	26 [23-31]
McCabe	7 (3.0%)	1 [1-2]
SAPSII	22 (9.5%)	59 [42-75]
Reason of ICU admission	3 (1.3%)	
Respiratory failure		120 (52)
Cardiac arrest		56 (24)
Circulatory failure		17 (7.4)
Kidney, hepatic and metabolic failure		15 (6.6)
Neurological failure		11 (4.8)
Post-operative		10 (4.4)
NMBA indication	1 (0.4%)	
ARDS		134 (58)
Hypothermia		43 (19)
P/V asynchrony		27 (12)
Other		27 (12)
Organ support during ICU stay		
Vasopressor	5 (2.2%)	185 (81)
RRT	9 (3.9%)	16 (7.2)
ECMO	5 (2.2%)	16 (7.0)

BMI: body mass index; ECMO: extracorporeal membrane oxygenation; ICU: intensive care unit ; IMV: invasive mechanical ventilation; NMBA: neuromuscular blocking agent; RRT: renal replacement therapy; SAPSII: simplified acute physiology score II

**Fig. 2:**
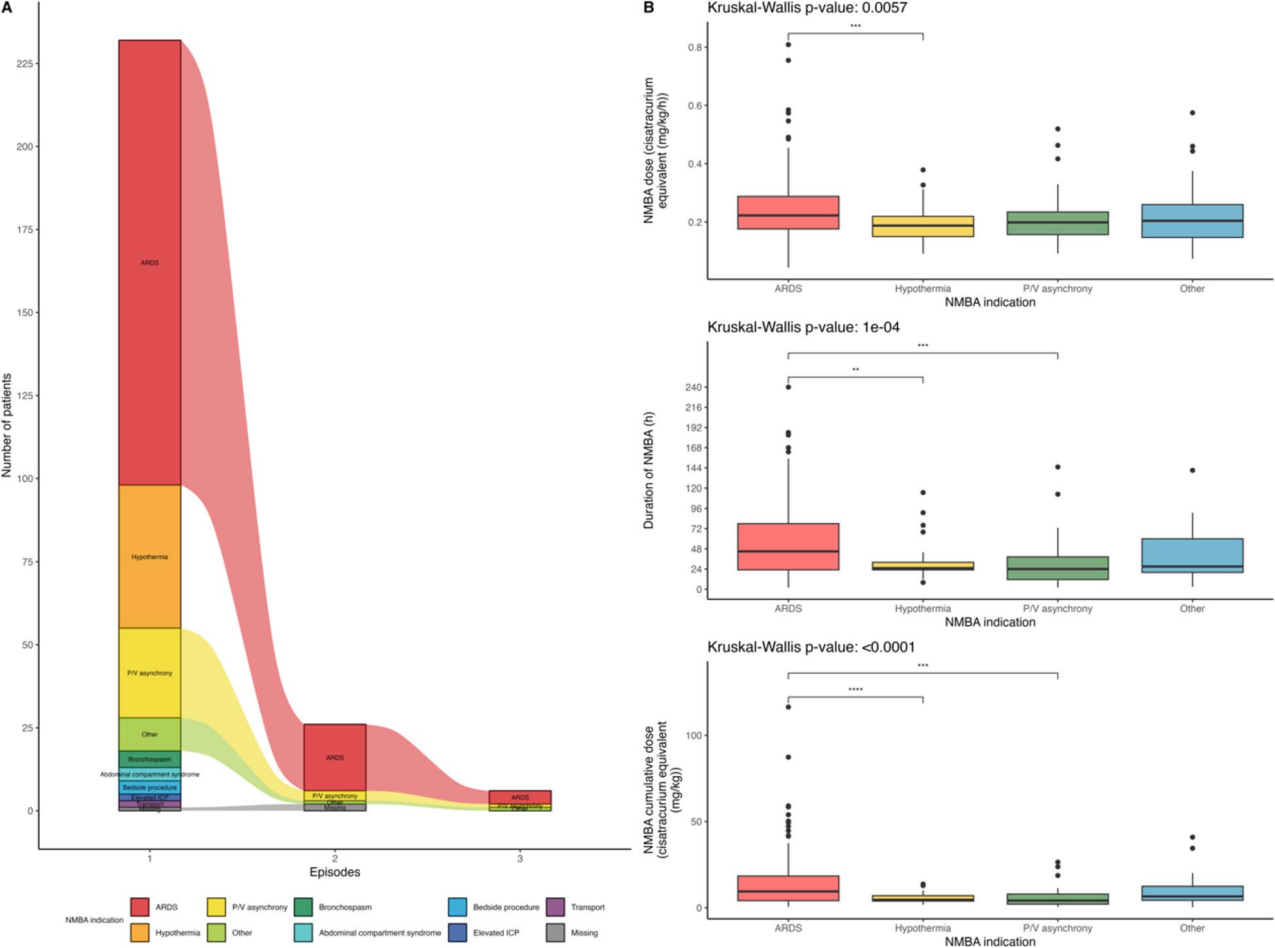
Indications of continuous NMBA infusion and duration and doses of infusion according to indications. **A** Indications of continuous NMBA infusion. **B** Hourly and cumulative doses and duration of continuous NMBA infusion according to indications. Overall statistical comparisons were performed with the Kruskall-Wallis test and post-hoc comparisons were performed with pairwise Wilcoxon tests with false-discovery rate corrected p-values (ns: p≥0.05, *p < 0.05, **p < 0.01, ***p < 0.001 and ****p < 10^-4^). ARDS: acute respiratory distress syndrome; ICP: intra-cranial pressure; P/V: patient-ventilator

Primary indications for NMBA infusion were ARDS (134, 58%), prevention of shivering during therapeutic hypothermia (43, 19%) and P/V asynchrony in patients without ARDS (27, 12%). The indication for NMBA remained consistent across episodes among the 20 (8.6%) of patients with 2 episodes of NMBA infusions and among the 6 (2.6%) of patients with 3 episodes of NMBA infusions (Fig. 2A). Given this consistency, all episodes were grouped for subsequent analyses.

NMBA infusion was initiated at a median of 0 [0-2] days after ICU admission. On the day of NMBA continuous infusion initiation, minimal PaO_2_/FiO_2_ was 114 [85-177] mmHg, and maximal PEEP and plateau pressure were 10 [6–12] cmH_2_O and 25 [20-29] cmH_2_O. In patients with ARDS as NMBA indication, PaO_2_/FiO_2_ was 97 [80-136] mmHg (100 (79%) of patients with PaO_2_/FiO_2_ < 150), PEEP was 10 [8–14] cmH_2_O and plateau pressure was 26 [21-29] cmH_2_O. Additionally in this subpopulation, prone position was performed in 39/134 (29%) on the day of NMBA initiation and in 64/134 (48%) during the entire ICU stay.

Cisatracurium was the most frequently used molecule (171, 74%) and median duration of NMBA infusion was 38 [22–71] hours. The total duration of infusion, hourly dosage, and cumulative doses varied significantly depending on the indication. ARDS was associated with the highest doses and longest infusion durations (Fig. 2B and Supplementary table 3).

### Description of NMBA protocols and monitoring

Among the 19 centers, 7 (37%) had a protocol for NMBA infusion monitoring, all of which were based on TOF monitoring. Of these, 5 (71%) were nurse-driven protocols. TOF targets ranged between 1 and 3, with a median monitoring frequency of 4 measurements per day (range: 2 to 12). Among centers without NBMA protocol, half reported the unavailability of TOF monitoring devices.

Out of the 232 patients who received continuous NMBA infusion, 111 (47.8%) had at least one TOF recording during the infusion, with a median of 10 [5–20] recordings per patient (5.7 [3.6–8.6] per 24 h) and a median TOF value of 0 [0-1]). Regarding the site of TOF monitoring, ulnar nerve stimulation was used exclusively in 50/111 (45%) patients, facial nerve stimulation in 40/111 (36%), both in 14/111 (13%) while information was missing in 7/111 (6%).

The median hourly dose of NMBA did not differ significantly between patients without TOF and those with TOF monitoring (0.20 [0.16–0.27] vs. 0.21 [0.17–0.26] mg/kg/h of cisatracurium equivalent, p = 0.5). However, patients with TOF monitoring had significantly longer infusion durations (53 [24–97] vs. 27 [21–59] hours p < 0.001) and, consequently, higher cumulative NMBA doses (9 [5–23] vs. 7 [4–14] mg/kg of cisatracurium equivalent, p < 0.001). TOF monitoring was also associated with more frequent dose adjustments (median 2 [0–5] vs. 0 [0–1], p < 0.001), primarily due to TOF values outside target range (p < 0.001). Dose adjustments due to P/V asynchrony or based solely on physician decision did not differ significantly (Table 2).

**Table 2:** Description of continuous NMBA administration

Variable	Missing	Overall	NMBA TOF monitoring		
		N = 232	No N = 121 (52%)	Yes N = 111 (48%)	Adj. p-value
Delay of NMBA administration (days)	1 (0.4%)	0.0 [0.0–2.0]	0.0 [0.0–1.0]	0.0 [0.0–2.0]	0.2
Number of episodes	0 (0%)	.0 [1.0–1.0]	.0 [1.0–1.0]	.0 [1.0–1.0]	0.064
Duration of NMBA infusion (hours)	0 (0%)	38 [22–72]	27 [21–59]	53 [24–100]	**<0.001**
Molecule	0 (0%)				0.065
Atracurium		61 (26)	38 (31)	23 (21)	
Cisatracurium		171 (74)	83 (69)	88 (79)	
NMBA dose (cisatracurium equivalent (mg/kg/hour))	1 (0.4%)	0.21 [0.17–0.27]	0.20 [0.16–0.27]	0.21 [0.17–0.27]	0.5
Cisatracurium dose (mg/kg/hour)	61 (26%)	0.21 [0.16–0.27]	0.20 [0.15–0.27]	0.21 [0.17–0.26]	0.5
Atracurium dose (mg/kg/hour)	172 (74%)	0.62 [0.53–0.81]	0.61[0.54–0.80]	0.64 [0.51–0.81]	0.6
NMBA cumulative dose (cisatracurium equivalent (mg/kg))	1 (0.4%)	8 [4–15]	7 [4–12]	9 [5–23]	**< 0.001**
Cisatracurium cumulative dose (mg/kg)	61 (26%)	8 [4–16]	7 [4–14]	9 [4–24]	**0.011**
Atracurium cumulative dose (mg/kg)	172 (74%)	23 [12–43]	15 [11–31]	34 [22–53]	**< 0.001**
Number of dose changes	0 (0%)	0 [0–2]	0 [0–1]	2 [0–5]	**< 0.001**
Increase	0 (0%)	0 [0–1]	0 [0–0]	1 [0–2]	**< 0.001**
Decrease	0 (0%)	0 [0–1]	0 [0–0]	1 [0–3]	**< 0.001**
Reason for dose change					
Asynchrony	0 (0%)	0 [0–0]	0 [0–0]	0 [0–0]	0.9
Outside TOF target	0 (0%)	0 [0–0]	0 [0–0]	0 [0–3]	**< 0.001**
Medical decision	0 (0%)	1 [0–2]	1 [0–2]	1 [0–1]	> 0.9
Monitoring protocol	0 (0%)	85 (37)	11 (9.1)	74 (67)	**< 0.001**

P-values were adjusted for multiple comparisons using the false discovery rate method. Adjusted p-values <0.05 are in bold

Adj. : adjusted; NMBA : neuromuscular blocking agents; TOF: train-of-four

### Patient outcomes

#### ICU mortality

**Fig. 3:**
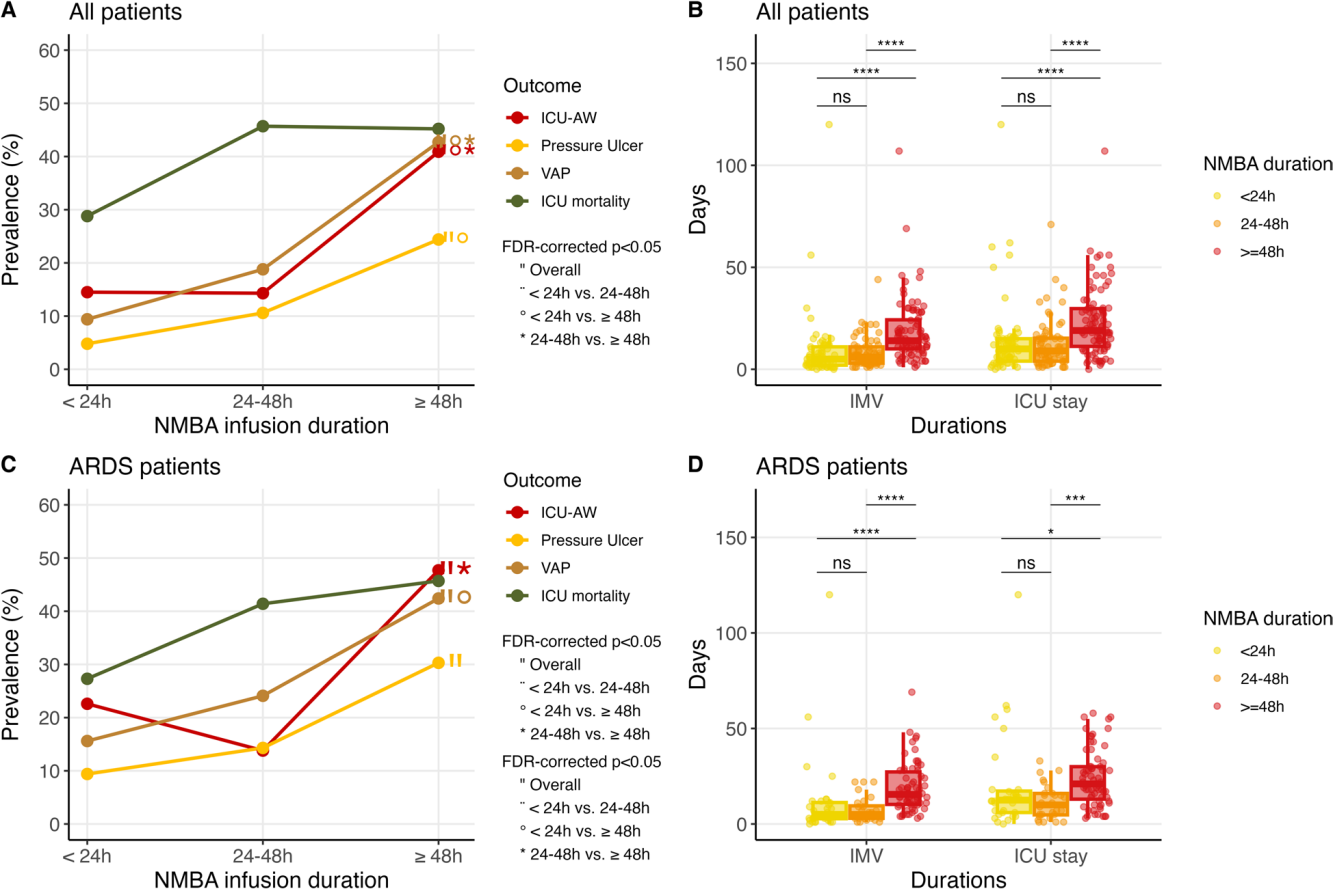
ICU and hospital outcomes according to continuous NMBA infusion duration. Outcomes according to continuous NMBA duration (less than 24h, 24h to 48h and more than 48h) in the whole population [upper panels, categorical (**A**) and continuous (**B**) outcomes] and in the ARDS population [lower panels, categorical (**C**) and continuous (**D**)]. Post-hoc comparisons were performed with pairwise Chi-square tests for the categorical outcome and pairwise Wilcoxon tests for the continuous outcome. All p-values were corrected for multiple comparisons using the false-discovery rate method (ns: p ≥ 0.05, *p < 0.05, **p < 0.01, ***p < 0.001 and ****p < 10^-4^). FDR: false-discovery rate; ICU: intensive care unit; ICU-AW: ICU-acquired weakness; IMV: invasive mechanical ventilation; NMBA: neuromuscular blocking agents; VAP: ventilator-associated pneumonia

ICU mortality was 93 (41%) in the whole population and 53 (40%) in patients receiving NMBA for ARDS. In both populations, no significant difference of mortality was found according to NMBA duration (<24 h, 24–48 h or 48 h) (Fig. 3A and C, Table 3 and Supplementary table 4).

**Table 3.** Relationships between NMBA infusion duration and ICU outcomes

Variable	Missing	Overall	NMBA infusion duration			
		N = 232	<24 h N = 67 (29%)	24–48 h N = 70 (30%)	> 48 h N = 95 (41%)	Adj. p-value
ICU-acquired weakness	19 (8.2%)	54 (25%)	9 (15%)	9 (14%)	36 (41%)	**< 0.001**
Pressure ulcer	17 (7.3%)	31(14%)	3 (4.8%)	7 (11%)	21 (24%)	**0.003**
Ventilator-associated pneumonia	10 (4.3%)	57 (26%)	6 (9.4%)	13 (19%)	38 (43%)	**< 0.001**
Tracheostomy	3 (1.3%)	17 (7.4%)	2 (3.0%)	3 (4.3%)	12 (13%)	**0.051**
Length of IMV (days)	11 (4.7%)	9 [4–16]	5 [2–11]	6 [3–11]	14 [10–25]	**< 0.001**
Length of ICU stay (days)	9 (3.9%)	13 [6–22]	10 [4–15]	9 [4–16]	19 [11-30]	**< 0.001**
ICU mortality	3 (1.3%)	93 (41%)	19 (29%)	32 (46%)	42 (45%)	0.068

P-values were adjusted for multiple comparisons using the false-discovery rate method. Adjusted p-values <0.05 are in bold

Adj. : adjusted; ICU: intensive care unit; IMV: invasive mechanical ventilation; NMBA: neuromuscular blocking agents

In multivariable analysis, BMI (hazard ratio (HR) 0.95 [95% confidence interval (95%CI)] [0.91, 0.99], p = 0.27, MacCabe score of 3 *vs.* 1 (HR 2.90 [1.24, 6.75], p = 0.014), SAPS II (HR 1.02 [1.01, 1.03], p = 0.006) and TOF monitoring (HR 0.55 [0.32, 0.95], p = 0.032) were associated with ICU mortality **(**Table 4 and Supplementary table 5). In patients with ARDS, only SAPSII (HR 1.02 [1.01, 1.03], p < 0.001) was associated with mortality while TOF monitoring was not (HR 0.59 [0.31, 1.11], p = 0.1) (Supplementary table 6).

**Table 4.** Multivariable analyses on ICU mortality, time to successful weaning, pressure ulcers, ventilator-associated pneumonia and ICU-acquired weakness

	In-ICU mortality			Successful weaning			Ventilator-associated pneumonia			Pressure ulcers			ICU-acquired weakness		
Variable	HR	95% CI	P	SHR	95% CI	P	OR	95% CI	P	OR	95% CI	P	OR	95% CI	P
Age (years)	1.01	0.98, 1.03	0.6	0.99	0.98, 1.01	0.2	0.99	0.97, 1.02	0.6						
BMI (kg/m^2^)	0.95	0.91, 0.99	**0.027**										1.05	1, 1.11	0.061
MacCabe															
1	Ref.	–	–	Ref.	–	–									
2	1.11	0.82, 1.49	0.5	0.70	0.43, 1.13	0.14									
3	2.80	1.21, 6.51	**0.017**	0.42	0.17, 1.01	0.052									
Type of admission															
Planned surgery													Ref.	–	–
Unplanned surgery													0.42	0.03, 7.22	0.5
Medical													0.50	0.11, 2.21	0.4
SAPSII	1.02	1.01, 1.03	**0.002**	0.98	0.97, 0.99	**<0.001**	0.99	0.97, 1.01	0.3	0.99	0.97, 1.01	0.5	1.00	0.98, 1.02	>0.9
Etiology															
Respiratory failure	Ref.	–	–	Ref.	–	–	Ref.	–	–				Ref.	–	–
Cardiac arrest	1.32	0.86, 2.00	0.2	0.65	0.34, 1.22	0.2	1.73	0.54, 5.55	0.4	1.31	0.34, 5.02	0.7	1.30	0.41, 4.08	0.7
Other	0.68	0.43, 1.07	0.10	1.05	0.66, 1.66	0.9	3.03	1.21, 7.59	**0.019**	1.62	0.53, 4.9	0.4	3.35	1.28, 8.74	**0.014**
ARDS															
No	Ref.	–	–				Ref.	–	–	Ref.	–	–	Ref.	–	–
Yes	0.93	0.59, 1.47	0.7				1.83	0.76, 4.44	0.2	3.81	1.08, 13.41	**0.038**	3.10	1.29, 7.49	**0.012**
Sedatives															
Midazolam				Ref.	–	–	Ref.	–	–				Ref.	–	–
Combination				0.56	0.3, 1.04	0.068	3.02	1.18, 7.76	**0.022**				1.23	0.46, 3.3	0.7
Propofol				1.30	0.73, 2.32	0.4	1.38	0.65, 2.95	0.4				0.64	0.26, 1.53	0.3
Opioids															
Sufentanil	Ref.	–	–										Ref.	–	–
Other	1.13	0.73, 1.75	0.6										0.46	0.16, 1.35	0.2
PEEP (cmH2O)										1.03	0.93, 1.14	0.6			
Plateau pressure (cmH2O)				0.98	0.95, 1.01	0.2									
PaO_2_/FiO_2_				1.00	1, 1.01	0.3									
TOF monitoring															
No	Ref.	–	–				Ref.	–	–	Ref.	–	–	Ref.	–	–
Yes	0.55	0.32, 0.95	**0.032**				1.33	0.61, 2.89	0.5	1.72	0.69, 4.3	0.2	2.90	1.2, 7.01	**0.018**
NMBA duration (days)	1.03	0.95, 1.10	0.5	0.83	0.76, 0.91	**<0.001**	1.44	1.16, 1.79	**0.001**	1.22	1.04, 1.44	**0.018**	1.18	0.97, 1.44	0.092
ECMO															
No										Ref.	–	–	Ref.	–	–
Yes										6.62	1.55, 28.25	**0.012**	5.68	1.64, 19.7	**0.007**
RRT															
No													Ref.	–	–
Yes													2.12	0.69, 6.52	0.2

Multivariable analyses on ICU-mortality,time to extubation, pressure ulcers, ventilator-associated pneumonia and ICU-acquired weakness

Hazard ratios (HR) from Cox proportional hazard regression models of the cumulative incidence of in-ICU mortality, sub-distribution hazards ratios (SHR) from Fine and Gray competing risk regression models of the time to extubation with death as a competing risk and odds ratios (OR) from binary logistic regression models of the occurrence of ventilator-associated pneumonia, pressure ulcers and ICU-acquired weakness

All independent variables with p < 0.20 on univariable analyses were included in the multivariable models

Only variables retained in at least in one outcome multivariable model are presented. Adjusted p-values <0.05 are in bold

95% CI: 95% confidence interval; ARDS: acute respiratory distress syndrome; BMI: body mass index; ECMO: extracorporeal membrane oxygenation; ICU: intensive care medicine; IMV: invasive mechanical ventilation; NMBA: neuromuscular blocking agent; P: p-value; PEEP: positive end-expiratory pressure; Ref: reference; RRT: renal replacement therapy; SAPSII: simplified acute physiology score II

#### Other potentially NMBA-related outcomes

ICU-acquired weakness was reported in 54 (25%) of patients with continuous NMBA infusion, pressure ulcers in 31 (14%) and ventilator-associated pneumonia in 57 (26%). Median invasive mechanical ventilation duration was 9 [4–16] days, and the median ICU length of stay was 13 [6–22] days.

Importantly, the incidence of ICU-acquired weakness, pressure ulcers, and ventilator-associated pneumonia was significantly higher in patients with prolonged infusion (>48h) than in those receiving either <24 h or 24–48 h (Fig. 3A and Table 3). Invasive mechanical ventilation duration and ICU length of stay were also longer with increasing NMBA continuous infusion duration, especially for >48h infusions (Fig. 3B and Table 3). Results were similar in patients with ARDS (Fig. 3C and D and Supplementary table 4).

In multivariable analyses, NMBA infusion duration was associated with lower cumulative incidence of weaning success (sub-distribution HR 0.83 [0.76, 0.91], p < 0.001 in all patients and 0.78 [0.7, 0.87], p < 0.001 in ARDS patients), higher odds of ventilator-associated pneumonia (odds ratio (OR) 1.44 [1.16, 1.79], p < 0.001 in all patients and 1.34 [1.07, 1.67], p = 0.011 in ARDS patients), and of pressure ulcers (only in all patients OR 1.22 [1.04, 1.44], p = 0.018, with OR 1.17 [0.93, 1.47], p = 0.2 in ARDS patients). The association of NMBA infusion duration with ICU-acquired weakness did not reach significance (OR 1.18 [0.97, 1.44], p = 0.092 in all patients and 1.13 [0.94, 1.37], p = 0.2 in ARDS patients). TOF monitoring was associated with higher odds of ICU-acquired weakness but only in the whole population (OR 2.90 [1.2, 7.01], p = 0.018 in all patients and 2.81 [0.89, 8.86], p = 0.078 in ARDS patients) (Table 4 and Supplementary tables 6–14).

## Discussion

Our multicenter prospective observational study provides an overview of NMBA use and monitoring in mechanically ventilated ICU patients across two European countries’ ICUs in recent years, just before the COVID-19 pandemic. The findings highlight key aspects of NMBA utilization, monitoring practices, and their association with patient outcomes.

We observed an overall prevalence of continuous NMBA infusion in mechanically ventilated patients of 10.3%, slightly lower than in earlier studies reporting prevalences ranging from 13% to 20% in mechanically ventilated ICU patients (13% in Arroliga *et al.* [8]; < 20% in Christensen *et al.* [9]) and also lower than in studies conducted during the same period in other countries (22.8% in Lin *et al.*[18]). A reduction in the use of NMBAs compared with previous years has already been observed [19]. A potential explanation for the fact that our study observed an even lower prevalence of NMBA infusion is the spreading of lighter sedation protocols [11] and increased awareness of NMBA-associated complications such as ICU-acquired weakness. It is highly unlikely that the publication of the ROSE study [3]—which found no survival benefit of NMBAs in ARDS patients—a few months prior to our data collection, had already influenced clinical practice.

As in earlier studies [8, 10], ARDS was the predominant indication for NMBA use. This reflects established practice, where NMBA is mostly used to improve oxygenation and minimize P/V asynchrony during severe ARDS, as demonstrated in landmark studies such as the ACURASYS trial [2]. It is possible that, despite the negative results of the ROSE trial [3], published only a few months before the start of inclusions, clinicians in our cohort may have nevertheless chosen to use NMBAs more frequently and for longer durations in this population. Beyond ARDS, NMBA are a therapeutic tool in a wider range of critical care scenarios, the principals being the prevention of shivering during therapeutic hypothermia in 16% of patients in our cohort and other respiratory indications (P/V asynchrony in patients without ARDS and persistent bronchospasm in status asthmaticus).

As expected, ARDS patients exhibited higher severity of pulmonary illness (lower PaO_2_/FiO_2_ and higher plateau pressures) compared to non-ARDS indications. Interestingly, we found greater prone positioning utilization in ARDS patients (29% on the day of NMBA initiation and in 48% during the entire ICU stay) compared to what was previously reported, such as the 16.3% observed in severe ARDS in the Lung SAFE study [9].

A duration of NMBA infusion exceeding 48 hours was associated with significantly higher rates of ventilator-associated pneumonia, ICU-acquired weakness and pressure ulcers. In contrast, short-term use (<48 h) was not associated with an increased incidence of these complications, suggesting a more favorable safety profile. In multivariable analysis, each additional day of NMBA exposure was linked to a lower likelihood of successful weaning from invasive mechanical ventilation and increased odds of ventilator-associated pneumonia and pressure ulcers. While the association with ICU-acquired weakness did not reach significance, the trend remains concerning. These complications are well-documented risks of extended NMBA exposure and contribute to increased morbidity in critically ill patients [20–22]. The duration of invasive mechanical ventilation and ICU stay was longer in patients receiving prolonged NMBA infusions, particularly those lasting more than 48 hours. This aligns with previous studies highlighting the risks of prolonged NMBA use, including delayed recovery [18]. These results are not unexpected since patients receiving deeper sedation and prolonged neuromuscular blockade tend to be the most severely ill. However, a question remains: do deeper sedation and NMBA use contribute directly to the morbidity and mortality of these patients, or are they merely markers of the severity of their illness? While we cannot draw such conclusions from our study, the observed associations underscore the need for cautious use and timely discontinuation of neuromuscular blockade whenever possible, especially after 48 hours of NMBA infusion.

The findings concerning the association between NMBA monitoring using TOF and outcomes are mixed and not straightforward to interpret. On the one hand, patients in whom TOF monitoring was implemented had nearly twice the duration of NMBA infusion and, as a direct result, received higher cumulative doses of NMBA. An association between TOF monitoring and higher cumulative NMBA doses, but not with infusion duration, has already been reported [23]. In multivariable analysis, we observed an association between TOF monitoring and an increased incidence of ICU-acquired weakness [24], potentially as a consequence of prolonged NMBA infusion which is associated with poorer outcomes. The longer infusion duration in the TOF monitoring group is unexpected, because TOF monitoring is generally recommended to reduce NMBA exposure and improve outcomes. However, given the observational design of our study, the heterogeneity of the population, and the variability in practices across centers, these associations between NMBA monitoring using TOF and outcomes must be interpreted with caution, as residual confounding and selection biases cannot be excluded.

A potential explanation is that the decision to implement TOF monitoring is more likely in patients with anticipated prolonged need for NMBA, such as thosewith severe ARDS. However, our results suggest that in real-world ICU settings, TOF monitoring may inadvertently contribute to NMBA overuse, possibly by prompting more frequent dose adjustments when TOF values deviate from predefined targets, thereby prolonging NMBA infusion duration. On the other hand, a surprising finding was the significant reduction in ICU mortality observed in multivariable analysis in the group with TOF monitoring, an observation that appears difficult to reconcile with the above associations. One possible explanation is that ICUs that adopt structured protocols such as TOF monitoring may deliver a generally higher quality of care, leading to improved outcomes, independent of the specific protocol itself and despite more prolonged exposure to NMBA. Overall, our study is coherent with the mixed results regarding the utility of TOF monitoring in reducing NMBA exposure and improving outcomes reported in previous studies. A randomized controlled trial comparing TOF assessment to clinical evaluation by bedside nurses found no difference in the mean total paralysis time, cumulative NMBA dose, and mean recovery time [15]. Another study evaluated a nurse-driven protocol for NMBA titration based on TOF monitoring in ARDS patients and found that nurses were able to decrease the amount of NMBA administered without significantly affecting the quality of the neuromuscular block achieved [16].

This study has several limitations. First, due to the observational design, analyses on outcomes should be viewed as exploratory. They only reveal statistical associations and cannot establish causality between NMBA infusion duration or TOF monitoring and patient outcomes. Second, because the study focused on NMBA use, the population was heterogeneous, particularly regarding admission diagnoses (respiratory failure, cardiac arrest, etc.) and NMBA indications (ARDS vs. non-ARDS). This heterogeneity complicates the interpretation of outcomes, especially survival. Although similar results were observed in analyses restricted to patients receiving NMBA for ARDS, differential effects of NMBA across patient subgroups cannot be excluded. Third, while the multicenter design enhances generalizability, variability in TOF monitoring protocols (some of which were nurse-driven and some not) and monitoring practices across centers may have introduced additional heterogeneity in the results. Finally, our data were collected 5–6 years ago, prior to the COVID-19 pandemic and the publication of large RCTs such as the ROSE trial [3], both of which may have influenced NMBA use and monitoring practices in current ICU settings. We also did not collect corticosteroid use which could impact ICU-acquired weakness in conjunction with NMBA [25], nor data on withdrawal and withholding of life-sustaining therapy, nor detailed the indication of NMBA within patients with ARDS (early protocolized NMBA used, rescue therapy or facilitation of prone positioning).

However, our study has notable strengths. It is one of the largest multicenter prospective investigations of NMBA use in the ICU, involving detailed data collection on indications, dosing, monitoring, and outcomes. To our knowledge, it is the only study that specifically addresses NMBA use both in the context of ARDS and in other clinical indications. Importantly, we report real-world clinical practice data, which differ from those of randomized controlled trials such as ACURASYS [2] and ROSE [3], where NMBA administration was protocolized and limited to 48 hours. In contrast, our findings show that in half of the patients, the duration of NMBA infusion exceeded 48 hours, and in a quarter of patients receiving TOF monitoring, it extended beyond 100 hours.

By highlighting the unintended consequences of TOF monitoring, this study provides valuable insights into the need for standardized and validated NMBA management protocols in critical care settings. Our findings raise important considerations for clinical practice. While TOF monitoring is widely recommended, its association with prolonged NMBA use, increased mechanical ventilation duration, and higher complication rates suggests that its implementation requires careful evaluation. Standardized TOF protocols and training for ICU teams are essential to ensure that monitoring achieves its intended goal of optimizing NMBA use without exacerbating complications.

Future research should focus on evaluating the efficacy of NMBA monitoring (with TOF or other techniques) in randomized controlled trials to determine its true role in NMBA management and patient outcomes; on the interaction between different ventilation strategies (e.g., prone versus supine ventilation) and the use of NMBAs; on the short term consequences of prolonged NMBA infusions; and on the long-term functional outcomes of ICU patients exposed to prolonged NMBA infusions, to understand the functional and quality-of-life implications of these practices.

## Conclusion

The CURATIV study provides valuable insights into the use and monitoring of neuromuscular blocking agents in mechanically ventilated intensive care unit patients across two European countries. Approximately one in ten such patients receive continuous neuromuscular blocking agents infusion, with acute respiratory distress syndrome remaining the most frequent indication. However, neuromuscular blocking agents are also used in a variety of other critical care contexts. Prolonged neuromuscular blocking agents exposure is associated with longer durations of mechanical ventilation and intensive care unit stay, as well as a higher risk of complications such as ventilator-associated pneumonia and pressure ulcers. The role of train-of-four monitoring in neuromuscular blocking agents use and patient outcomes remains unclear, with potentially both beneficial and unintended effects. These findings highlight the need for standardized neuromuscular blocking agents management protocols and further randomized trials to better define the role of monitoring strategies in improving patient outcomes.

## Supplementary Information

Additional file 1.

## Data Availability

The study data will be made available upon reasonable request to the Research Commission of the French Intensive Care Society.

## Abbreviations

ARDS: Acute respiratory distress syndrome
BMI: Body mass index
BSAS: Bedside shivering assessment scale
HR: Hazard ratio
ICU: Intensive care unit
ICU-AW: ICU-acquired weakness
IMV: Invasive mechanical ventilation
IQR: Interquartile range
MICE: Multiple imputation by chained equation
NMBA: Neuromuscular blocking agents
OR: Odds ratio
PEEP: Positive end-expiratory pressure
P/V: Patient-ventilator
PU: Pressure ulcer
RRT: Renal replacement therapy
SAPSII: Simplified acute physiology score
SRLF: Société de réanimation de langue française
SHR: Subdistribution hazard ratio
VAP: Ventilator-associated pneumonia
TOF: Train-of-four
