# Supplementary material for: Neuromuscular blockade and their monitoring in the intensive care unit: a multicenter observational prospective study
Source: Ann Intensive Care. 2025 Oct 22;15:167. doi: 10.1186/s13613-025-01591-4 (PMC12546236; doi:10.1186/s13613-025-01591-4)
Supplement: Supplementary file 1 — Additional file 1. [file 13613_2025_1591_MOESM1_ESM.docx]

**SUPPLEMENTARY MATERIAL**

**Neuromuscular blockade and their monitoring in the intensive care unit: a multicenter observational prospective study**

**Authors**

Bertrand Hermann^1,2^, Guillaume Decormeille^3^, Tiphanie Gobé^4^, Nathanaël Mangeard^4^, Adel Maamar^4^, Saria Sayadi^5^, Bénédicte Pernod^1^, Nadine Robquin^6^, Jean-Pierre Ponthus^6^, Sophie Le Potier^7^, Pierre Bouju^7^, Angélique Balabanian^8^, Antoine Frouin^9^, Sébastien Moschietto^9♱^, Gwenaelle Jacq^10^, Emeline Villemont^11^, Clémence Houbé^12^, Anaïs Queyreau^12^, Célina Morand^13^, Florence Boissier^13^, Jean-Baptise Lascarrou^14^, Sabine Valera^15^, Sami Hraiech^15^, Laure Clouet^16^, Gaël Piton^16^, Cindérella Noël^17^, Anne Joosten^17^, Cécilia Tabra Osorio^18^, Adrien Constan^18^, Jérôme Cecchini^18^, Gwennaelle Mercier^19^, Arnaud Bruyneel^20^, Chloé Villamaux^21^, François Pousset^21^, Nicholas Heming^22^, Laurent Poiroux^23^, Jean-François Llitjos^24^ and Saber Davide Barbar^11,25^ on behalf of the SRLF Trial Group

1. Service de Médecine intensive-Réanimation, Hôpital Européen Georges Pompidou, Assistance Publique – Hôpitaux de Paris (AP-HP), France

2. Université Paris Cité, INSERM 1266 , Institute of Psychiatry and Neurosciences of Paris (IPNP), INSERM UMR 1266, Paris, France, F-75006 Paris.

3. Service de Réanimation Médicale, CHU Toulouse, France

4. Service de Réanimation Médicale, CHU Pontchaillou, Rennes, France

5. Service de Médecine intensive-Réanimation, Hôpital Ambroise Paré, Assistance Publique – Hôpitaux de Paris (AP-HP), France

6. Service de Médecine intensive-Réanimation, Hôpital Lucie et Raymond Aubrac, Villeneuve-Saint-Georges, France

7. Service de Réanimation Polyvalente, CH de Lorient, France

8. Service de Réanimation Médico-Chirurgicale, Hôpital René Dubos, Pontoise, France

9. Service de Réanimation Polyvalente, CH d’Avignon, France

10. Service de Médecine intensive-Réanimation, CH André Mignot, Le Chesnay, France

11. Division of Anesthesia, Critical Care, Pain and Emergency Medicine, Nîmes University Hospital, University of Montpellier, Nîmes, France

12. Service de Réanimation Polyvalente Adulte, Hôpital Necker, Assistance Publique – Hôpitaux de Paris (AP-HP), France

13. Service de Médecine intensive-Réanimation, CHU Poitiers, France

14. Service de Médecine intensive-Réanimation, CHU Nantes, France

15. Service de Médecine intensive-Réanimation, Assistance Publique – Hôpitaux de Marseille, France

16. Service de Médecine intensive-Réanimation, CHU Besançon, France

17. Unité de Soins Intensifs, Hôpital Civil Marie Curie, Lodelinsart, Belgique

18. Service de Médecine intensive – Réanimation, Centre hospitalier Intercommunal de Créteil, France

19. Service de Soins Intensifs, Hôpital Erasme, HUB, Bruxelles, Belgique

20. Health Economics, Hospital Management and Nursing Research Dept, School of Public Health, Université Libre de Bruxelles, Belgium

21. Service de Réanimation Polyvalente, CH Mayotte, France

22. Service de Médecine intensive-Réanimation, Hôpital Raymond Poincaré, Assistance Publique – Hôpitaux de Paris (AP-HP), France

23. Service de Médecine intensive – Réanimation, CHU d’Angers, France

24. Laboratoire BioMérieux, Craponne, France

25. UR‑UM103 IMAGINE, University of Montpellier, Nimes University Hospital, Nîmes, France.

♱ deceased

**Table of content**

1. **Definitions of outcomes…………………………………………………………….page 3**
2. **Description of NMBA administered in single bolus……………………………...page 4**
3. **Description of NMBA administered in continuous infusion……………….........page 6**
4. **Relationship between ICU outcomes and NMBA duration in patients receiving NMBA for acute respiratory distress syndrome………………………………....page 7**
5. **Univariable and multivariable analyses of ICU outcomes…………………........page 8**

1. **Definitions of outcomes**

Ventilator-associated pneumonia was diagnosed as the initiation by the physician in charge of a new antimicrobial therapy prompted by two signs among fever, leukocytosis, purulent tracheal secretions, rising oxygen requirements and a new or worsening infiltrate on chest X-ray. These data were not collected and no central adjudication committee was implemented.

P/V asynchrony was defined as a persistent mismatch between patient effort and ventilator support, identified on ventilator waveforms and clinical signs (e.g., distress, accessory muscle use, and increasing oxygen requirements), but in the absence of persistent severe hypoxemia (PaO₂/FiO₂ <150 mmHg), which defines ARDS requiring NMBA use in the ACURASYS trial [2].

ICU-acquired weakness was defined as a diffuse, symmetric weakness involving limb, respiratory (including diaphragm), axial, and sometimes bulbar muscles, not explained by pre-existing neuromuscular disorders or other plausible causes, with a Medical Research Council score <48/60 performed by the physician in charge or by a physiotherapist.

Pressure ulcers were defined as any skin lesion caused by sustained pressure, documented in the medical record by the attending physician or nursing staff, and when available, staged according to the internationally recognized EPUAP/NPUAP classification system (Stage I–IV).

1. **Description of NMBA administered in single bolus**

A total of 95 patients out of the 2248 who underwent mechanical ventilation over the study period received NMBA in bolus, that is a prevalence of NMBA bolus infusion of 4.3%.

A total of 107 boluses were administered to 95 patients. A description of the population and NMBA administration characteristics is presented in **Supplementary table 1** and **Supplementary table 2** below, respectively.

| **Variable** | **Bolus population N = 95^1^** | **Missing N (%)** |
| --- | --- | --- |
| **Age (years)** | 62 [52-69] | 2 (2.1%) |
| **Gender** |  | 0 (0%) |
| Female | 32 (34) |  |
| Male | 63 (66) |  |
| **BMI (kg/m2)** | 26 [23-32] | 6 (6.3%) |
| **McCabe** | 1 [1-2] | 4 (4.2%) |
| **SAPS II** | 54 [41-69] | 11 (12%) |
| **Reason of ICU admission** |  | 2 (2.1%) |
| Respiratory failure | 43 (46) |  |
| Cardiac arrest | 12 (13) |  |
| Circulatory failure | 13 (14) |  |
| Kidney, hepatic and metabolic failure | 8 (8.6) |  |
| Neurological failure | 10 (11) |  |
| Post-operative | 7 (7.5) |  |
| **Delay of NMBA administration (days)** | 1.0 [0.0-2.0] | 2 (2.1%) |
| **Length of MV (days)** | 7 [3-15] | 4 (4.2%) |
| **Length of ICU stay (days)** | 14 [8-21] | 3 (3.2%) |
| **ICU survival** | 63 (67) | 1 (1.1%) |
| **Length of hospital stay (days)** | 26 [15-40] | 16 (17%) |
| **Hospital survival** | 60 (65) | 3 (3.2%) |
| ^1^n (%); Median [25%-75%] | | |

**Supplementary table 1. Characteristics of the population receiving NMBA bolus infusions**.

ARDS : acute respiratory distress syndrome; BMI : body mass index; ICP : intracranial hypertension; ICU : intensive care unit; MV: mechanical ventilation; NMBA : neuromuscular blocking agents; P/V : patient/ventilator asynchrony; SAPS II : simplified acute physiology score II.

| **Variable** | **NMBA bolus episodes N = 107^1^** | **Missing** |
| --- | --- | --- |
| **Number of episodess** |  | 0 (0%) |
| 1 | 95 (89) |  |
| 2 | 10 (9.3) |  |
| 3 | 1 (0.9) |  |
| 4 | 1 (0.9) |  |
| **Molecule** |  | 0 (0%) |
| Atracurium | 47 (44) |  |
| Cisatracurium | 60 (56) |  |
| **NMBA indication** |  | 0 (0%) |
| Abdominal Compartment Syndrome | 1 (0.9) |  |
| ARDS | 21 (20) |  |
| Elevated ICP | 2 (1.9) |  |
| Endoscopic procedure | 36 (34) |  |
| Hypothermia | 4 (3.7) |  |
| Neurophysiological exam | 10 (9.3) |  |
| Other | 4 (3.7) |  |
| P/V asynchrony | 17 (16) |  |
| Surgical procedure | 5 (4.7) |  |
| Transport | 7 (6.5) |  |
| **NMBA dose (cisatracurium equivalent, mg/kg))** | 0.22 [0.17-0.29] | 11 (10%) |
| Atracurium dose (mg/kg) | 0.63 [0.50-0.76] |  |
| Cisatracurium dose (mg/kg) | 0.22 [0.17-0.29] |  |
| ^1^n (%); Median [25%-75%] | | |

**Supplementary table 2. Characteristics of the NMBA bolus infusions episodes.**

ARDS : acute respiratory distress syndrome; ICP : intracranial hypertension; NMBA : neuromuscular blocking agents; P/V : patient/ventilator asynchrony.

1. **Description of continuous NMBA infusion**

| **Variable** | **ARDS**  **N = 134** | **Hypothermia**  **N = 43** | **P/V asynchrony**  **N = 27** | **Other**  **N = 27** | **Adj.  p-value** |
| --- | --- | --- | --- | --- | --- |
| **Continuous NMBA administration** |  |  |  |  |  |
| **Delay of NMBA administration (days)** | 0 [0-2] | 0 [0-0] | 1 [0-2] | 1 [0-3] | **<0.001** |
| **Number of episodes** | 1 [1-1] | 1 [1 -1] | 1 [1-1] | 1 [1-1] | 0.2 |
| **Duration of NMBA (h)** | 54 [24-88] | 25 [23-39] | 24 [11-41] | 30 [19-65] | **<0.001** |
| **Molecule** |  |  |  |  | **0.004** |
| Atracurium | 29 (22) | 15 (35) | 14 (52) | 3 (11) |  |
| Cisatracurium | 105 (78) | 28 (65) | 13 (48) | 24 (89) |  |
| **NMBA dose (cisatracurium equivalent (mg/kg/h))** | 0.22  [0.18-0.29] | 0.19  [0.15-0.22] | 0.19  [0.15-0.23] | 0.20  [0.14-0.27] | **0.007** |
| **Cisatracurium dose (mg/kg/h)** | 0.22  [0.18-0.29] | 0.18  [0.15-0.22] | 0.16  [0.14-0.20] | 0.19  [0.14-0.25] | **0.007** |
| **Atracurium dose (mg/kg/h)** | 0.68  [0.57-0.89] | 0.57  [0.48-0.66] | 0.62  [0.56-0.72] | 0.99  [0.61-1.72] | 0.12 |
| **NMBA cumulative dose (cisatracurium equivalent (mg/kg))** | 10 [6-23] | 5 [4-8] | 4 [2-8] | 7 [4-16] | **<0.001** |
| **Cisatracurium cumulative dose (mg/kg)** | 9 [5-24] | 5 [3-8] | 5 [2-8] | 6 [4-14] | **0.001** |
| **Atracurium cumulative dose (mg/kg)** | 41 [21-52] | 15 [12-24] | 12 [4-25] | 3 [22-103] | **0.006** |
| **Number of dose changes** | 1 [0-3] | 0 [0-0] | 0 [0-1] | 0 [0-2] | **<0.001** |
| Increase | 0 [0-1] | 0 [0-0] | 0 [0-0] | 0 [0-1] | **<0.001** |
| Decrease | 0 [0-1] | 0 [0-0] | 0 [0-1] | 0 [0-0] | **0.001** |
| **Reason for dose change** | 0 [0-1] | 0 [0-0] | 0 [0-0] | 0 [0-0] |  |
| Asynchrony | 0 [0-1] | 0 [0-0] | 0 [0-0] | 0 [0-0] | **0.039** |
| Outside TOF target | 1 [0-2] | 0 [0-1] | 1 [0-2] | 0 [0-2] | **0.008** |
| Medical decision |  |  |  |  | 0.2 |
| **NMBA monitoring** |  |  |  |  |  |
| **Monitoring protocol** | 61 (46) | 5 (12) | 6 (22) | 13 (48) | **<0.001** |
| **TOF monitoring** | 75 (56) | 15 (35) | 7 (26) | 13 (48) | **0.013** |
| **Number of TOF monitoring** | 3  [0-12] | 0  [0-6] | 0  [0-1] | 0  [0-7] | **0.010** |
| **Frequency of TOF monitoring (/24h)** | 1.3  [0.0-6.3] | 0.0  [0.0-4.6] | 0.0  [0.0-4.0] | 0.0  [0.0-5.3] | **0.043** |
| **Site of TOF monitoring** |  |  |  |  |  |
| Ulnar nerve (adductor pollicis) | 41 (31) | 11 (26) | 3 (11) | 8 (30) | 0.2 |
| Facial nerve (orbicularis oculari) | 41 (31) | 2 (4.7) | 5 (19) | 6 (22) | **0.009** |
| **Median TOF values** | 0 [0-1] | 0 [0-0] | 2 [0-4] | 0 [0-4] | 0.10 |

**Supplementary table 3. Characteristics of continuous NMBA infusions administration and monitoring according to NMBA indication.**Abbreviations: Adj. : Adjusted; ARDS: acute respiratory distress syndrome; ICU: intensive care medicine; NMBA: neuromuscular blocking agent; P/V: patient-ventilator; TOF: train-of-four.

1. **Relationship between ICU outcomes and NMBA duration in patients receiving NMBA for acute respiratory distress syndrome**

|  | | **Overall** | **NMBA infusion duration** | | |  |
| --- | --- | --- | --- | --- | --- | --- |
| **Variable** | **Missing (N (%))** | **N=134** | **<24h N=33 (25%)** | **24-48h N=29 (22%)** | **>48h N=72 (54%)** | **Adj.  p-value** |
| **ICU-acquired weakness** | **9 (6.7%)** | **42 (34)** | **7 (23)** | **4 (14)** | **31 (48)** | **0.004** |
| **Pressure ulcer** | **8 (6.0%)** | **27 (21)** | **3 (9.4)** | **4 (14)** | **20 (30)** | **0.049** |
| **Ventilator-associated pneumonia** | **7 (5.2%)** | **40 (31)** | **5 (16)** | **7 (24)** | **28 (42)** | **0.030** |
| **Tracheostomy** | **2 (1.5%)** | **8 (6.1)** | **1 (3.0)** | **0 (0)** | **7 (10)** | **0.2** |
| **Length of IMV (days)** | **8 (6.0%)** | **11 [5-19]** | **5 [3-12]** | **5 [3-10]** | **16 [10-28]** | **<0.001** |
| **Length of ICU stay (days)** | **7 (5.2%)** | **16 [7-28]** | **13 [6-18]** | **10 [5-16]** | **21 [12-30]** | **<0.001** |
| **ICU mortality** | **2 (1.5%)** | **53 (40)** | **9 (27)** | **12 (41)** | **32 (46)** | **0.2** |

**Supplementary table 4. Relationship between ICU outcomes and NMBA duration in patients receiving NMBA for acute respiratory distress syndrome.**

P-values were corrected for multiple comparisons using the false-discovery rate method.
Abbreviations : Adj. : adjusted p-values; ICU: intensive care unit; IMV: invasive mechanical ventilation; NMBA: neuromuscular blocking agents.

1. **Univariable and multivariable analyses of ICU outcomes**

**Supplementary table 5 & 6. Factors associated with in-ICU mortality.**

|  | **Univariable** | | | **Multivariable** | | |
| --- | --- | --- | --- | --- | --- | --- |
| **Variable** | **HR** | **95% CI** | **P** | **HR** | **95% CI** | **P** |
| **Age (years)** | 1.02 | 1.00, 1.04 | 0.11 | 1.01 | 0.98, 1.03 | 0.6 |
| **Gender** |  |  |  |  |  |  |
| Woman | — | — |  |  |  |  |
| Man | 0.85 | 0.58, 1.25 | 0.4 |  |  |  |
| **BMI (kg/m²)** | 0.95 | 0.91, 1.00 | **0.036** | 0.95 | 0.91, 0.99 | **0.027** |
| **MacCabe** |  |  |  |  |  |  |
| 1 | — | — |  | — | — |  |
| 2 | 1.28 | 0.85, 1.92 | 0.2 | 1.11 | 0.82, 1.49 | 0.5 |
| 3 | 2.24 | 1.11, 4.51 | **0.025** | 2.80 | 1.21, 6.51 | **0.017** |
| **Type of admission** |  |  |  |  |  |  |
| Planned surgery | — | — |  |  |  |  |
| Unplanned surgery | 0.82 | 0.15, 4.42 | 0.8 |  |  |  |
| Medical | 1.70 | 0.63, 4.58 | 0.3 |  |  |  |
| **SAPSII** | 1.02 | 1.01, 1.03 | **<0.001** | 1.02 | 1.01, 1.03 | **0.002** |
| **Etiology** |  |  |  |  |  |  |
| Respiratory failure | — | — |  | — | — |  |
| Cardiac arrest | 1.56 | 1.10, 2.23 | **0.014** | 1.32 | 0.86, 2.00 | 0.2 |
| Other | 0.71 | 0.45, 1.12 | 0.14 | 0.68 | 0.43, 1.07 | 0.10 |
| **ARDS** |  |  |  |  |  |  |
| No | — | — |  | — | — |  |
| Yes | 0.71 | 0.49, 1.03 | 0.074 | 0.93 | 0.59, 1.47 | 0.7 |
| **Sedatives** |  |  |  |  |  |  |
| Midazolam | — | — |  |  |  |  |
| Combination | 0.99 | 0.53, 1.85 | >0.9 |  |  |  |
| Propofol | 1.18 | 0.65, 2.12 | 0.6 |  |  |  |
| **Opioids** |  |  |  |  |  |  |
| Sufentanil | — | — |  | — | — |  |
| Other | 1.52 | 0.99, 2.34 | 0.055 | 1.13 | 0.73, 1.75 | 0.6 |
| **PEEP (cmH2O)** | 0.99 | 0.94, 1.04 | 0.8 |  |  |  |
| **Plateau pressure (cmH2O)** | 1.01 | 0.99, 1.04 | 0.3 |  |  |  |
| **PaO₂/FiO₂** | 1.00 | 1.00, 1.00 | 0.7 |  |  |  |
| **TOF monitoring** |  |  |  |  |  |  |
| No | — | — |  | — | — |  |
| Yes | 0.47 | 0.26, 0.85 | **0.013** | 0.55 | 0.32, 0.95 | **0.032** |
| **NMBA duration (days)** | 0.92 | 0.85, 1.01 | 0.075 | 1.03 | 0.95, 1.10 | 0.5 |
| **NMBA dose (cis. eq.)** | 2.29 | 0.36, 14.7 | 0.4 |  |  |  |
| **Vasopressor** |  |  |  |  |  |  |
| No | — | — |  |  |  |  |
| Yes | 1.12 | 0.63, 2.02 | 0.7 |  |  |  |
| **ECMO** |  |  |  |  |  |  |
| No | — | — |  |  |  |  |
| Yes | 0.86 | 0.38, 1.99 | 0.7 |  |  |  |
| **RRT** |  |  |  |  |  |  |
| No | — | — |  |  |  |  |
| Yes | 0.88 | 0.41, 1.92 | 0.8 |  |  |  |

**Supplementary table 5. Factors associated with In-ICU mortality in the whole population**

Univariable and multivariable Cox proportional hazard regression models were fitted to model the cumulative incidence of in-ICU mortality. All univariable and multivariable Cox models were clustered on centers, with bootstrapped robust confidence interval computation (1000 boot replicates). All independent variables with p<0.20 on univariable analyses were included in the multivariable model.

Abbreviations: ARDS: acute respiratory distress syndrome; BMI: body mass index; CI: confidence interval; ECMO: extracorporeal membrane oxygenation; HR; hazard ratio; ICU: intensive care medicine; IMV: invasive mechanical ventilation; NMBA: neuromuscular blocking agent; PEEP: positive end-expiratory pressure; RRT: renal replacement therapy; SAPSII: simplified acute physiology score II.

|  | **Univariable** | | | **Multivariable** | | |  |
| --- | --- | --- | --- | --- | --- | --- | --- |
| **Variable** | **HR** | **95% CI** | **P** | **HR** | **95% CI** | **P** |  |
| **Age (years)** | 1.03 | 1.00, 1.06 | **0.067** | 1.02 | 0.99, 1.05 | 0.2 |  |
| **Gender** |  |  |  |  |  |  |  |
| Woman | — | — |  |  |  |  |  |
| Man | 0.98 | 0.57, 1.70 | >0.9 |  |  |  |  |
| **BMI (kg/m²)** | 0.94 | 0.87, 1.01 | **0.093** | 0.95 | 0.88, 1.03 | **0.2** |  |
| **MacCabe** |  |  |  |  |  |  |  |
| 1 | — | — |  | — | — |  |  |
| 2 | 1.25 | 0.77, 2.01 | 0.4 | 1.22 | 0.89, 1.68 | 0.2 |  |
| 3 | 2.62 | 0.99, 6.92 | **0.053** | 2.71 | 0.74, 9.93 | **0.13** |  |
| **SAPSII** | 1.02 | 1.01, 1.03 | **<0.001** | 1.02 | 1.01, 1.04 | **0.003** |  |
| **Etiology** |  |  |  |  |  |  |  |
| Respiratory failure | — | — |  | — | — |  |  |
| Cardiac arrest | 0.83 | 0.34, 2.02 | 0.7 | 1.08 | 0.54, 2.16 | 0.8 |  |
| Other | 0.44 | 0.21, 0.91 | **0.028** | 0.57 | 0.27, 1.23 | **0.15** |  |
| **Sedatives** |  |  |  |  |  |  |  |
| Midazolam | — | — |  |  |  |  |  |
| Combination | 1.21 | 0.64, 2.29 | 0.6 |  |  |  |  |
| Propofol | 0.85 | 0.33, 2.18 | 0.7 |  |  |  |  |
| **Opioids** |  |  |  |  |  |  |  |
| Sufentanil | — | — |  | — | — |  |  |
| Other | 1.67 | 1.00, 2.78 | **0.048** | 1.07 | 0.57, 2.01 | 0.8 |  |
| **PEEP (cmH2O)** | 1.02 | 0.94, 1.11 | 0.6 |  |  |  |  |
| **Plateau pressure (cmH2O)** | 1.01 | 0.97, 1.05 | 0.7 |  |  |  |  |
| **PaO₂/FiO₂** | 1.00 | 0.99, 1.00 | 0.5 |  |  |  |  |
| **TOF monitoring** |  |  |  |  |  |  |  |
| No | — | — |  | — | — |  |  |
| Yes | 0.59 | 0.30, 1.15 | **0.12** | 0.59 | 0.31, 1.11 | **0.10** |  |
| **NMBA duration (days)** | 0.97 | 0.86, 1.09 | 0.6 |  |  |  |  |
| **NMBA dose (cis. eq.)** | 2.21 | 0.34, 14.5 | 0.4 |  |  |  |  |
| **Vasopressor** |  |  |  |  |  |  |  |
| No | — | — |  |  |  |  |  |
| Yes | 1.28 | 0.55, 2.96 | 0.6 |  |  |  |  |
| **ECMO** |  |  |  |  |  |  |  |
| No | — | — |  |  |  |  |  |
| Yes | 0.96 | 0.40, 2.28 | >0.9 |  |  |  |  |
| **RRT** |  |  |  |  |  |  |  |
| No | — | — |  |  |  |  |  |
| Yes | 0.77 | 0.29, 2.02 | 0.6 |  |  |  |  |
| **Prone position** | |  |  |  |  |  |  |
| No | | — | — |  | — | — |  |
| Yes | | 1.03 | 0.65, 1.63 | 0.9 | 1.32 | 0.76, 2.30 | 0.3 |

**Supplementary table 6. Factors associated with In-ICU mortality in ARDS patients.**

Univariable and multivariable Cox proportional hazard regression models were fitted to model the cumulative incidence of in-ICU mortality. All univariable and multivariable Cox models were clustered on centers, with bootstrapped robust confidence interval computation (1000 boot replicates). All independent variables with p<0.20 on univariable analyses were included in the multivariable model.

Abbreviations: ARDS: acute respiratory distress syndrome; BMI: body mass index; CI: confidence interval; ECMO: extracorporeal membrane oxygenation; HR; hazard ratio; ICU: intensive care medicine; IMV: invasive mechanical ventilation; NMBA: neuromuscular blocking agent; PEEP: positive end-expiratory pressure; RRT: renal replacement therapy; SAPSII: simplified acute physiology score II.

**Supplementary table 7 & 8. Factors associated with cumulative incidence of successful weaning**

|  | **Univariable** | | | **Multivariable** | | |
| --- | --- | --- | --- | --- | --- | --- |
| **Variable** | **SHR** | **95% CI** | **P** | **SHR** | **95% CI** | **P** |
| **Age (years)** | 0.99 | 0.97, 1 | **0.023** | 0.99 | 0.98, 1.01 | 0.2 |
| **Gender** |  |  |  |  |  |  |
| Woman | - | - |  |  |  |  |
| Man | 1.11 | 0.73, 1.69 | 0.625 |  |  |  |
| **BMI (kg/m²)** | 1.00 | 0.97, 1.03 | 0.868 |  |  |  |
| **MacCabe** |  |  |  |  |  |  |
| 1 | - | - |  | - | - |  |
| 2 | 0.81 | 0.53, 1.24 | 0.335 | 0.70 | 0.43, 1.13 | 0.14 |
| 3 | 0.45 | 0.19, 1.03 | 0.060 | 0.42 | 0.17, 1.01 | 0.052 |
| **Type of admission** |  |  |  |  |  |  |
| Planned surgery | - | - |  |  |  |  |
| Unplanned surgery | 1.16 | 0.39, 3.41 | 0.793 |  |  |  |
| Medical | 0.88 | 0.44, 1.79 | 0.727 |  |  |  |
| **SAPSII** | 0.98 | 0.97, 0.99 | **<0.001** | 0.98 | 0.97, 0.99 | **<0.001** |
| **Etiology** |  |  |  |  |  |  |
| Respiratory failure | - | - |  | - | - |  |
| Cardiac arrest | 1.04 | 0.63, 1.72 | 0.884 | 0.65 | 0.34, 1.22 | 0.2 |
| Other | 1.51 | 0.99, 2.32 | 0.056 | 1.05 | 0.66, 1.66 | 0.9 |
| **ARDS** |  |  |  |  |  |  |
| No | - | - |  |  |  |  |
| Yes | 0.98 | 0.67, 1.45 | 0.929 |  |  |  |
| **Sedatives** |  |  |  |  |  |  |
| Midazolam | - | - |  | - | - |  |
| Combination | 0.60 | 0.32, 1.12 | 0.109 | 0.56 | 0.3, 1.04 | 0.068 |
| Propofol | 1.05 | 0.63, 1.75 | 0.851 | 1.30 | 0.73, 2.32 | 0.4 |
| **Opioids** |  |  |  |  |  |  |
| Sufentanil | - | - |  |  |  |  |
| Other | 0.96 | 0.33, 2.84 | 0.945 |  |  |  |
| **PEEP (cmH2O)** | 0.98 | 0.93, 1.03 | 0.356 |  |  |  |
| **Plateau pressure (cmH2O)** | 0.97 | 0.94, 1 | **0.027** | 0.98 | 0.95, 1.01 | 0.2 |
| **PaO₂/FiO₂** | 1.00 | 1, 1.01 | 0.113 | 1.00 | 1, 1.01 | 0.3 |
| **TOF monitoring** |  |  |  |  |  |  |
| No | - | - |  |  |  |  |
| Yes | 1.36 | 0.72, 2.6 | 0.347 |  |  |  |
| **NMBA duration (days)** | 0.86 | 0.78, 0.94 | **0.001** | 0.83 | 0.76, 0.91 | **<0.001** |
| **NMBA dose (cis. eq.)** | 0.80 | 0.11, 5.64 | 0.825 |  |  |  |
| **Vasopressor** |  |  |  |  |  |  |
| No | - | - |  |  |  |  |
| Yes | 0.92 | 0.58, 1.44 | 0.706 |  |  |  |
| **ECMO** |  |  |  |  |  |  |
| No | - | - |  |  |  |  |
| Yes | 0.74 | 0.38, 1.47 | 0.391 |  |  |  |
| **RRT** |  |  |  |  |  |  |
| No | - | - |  |  |  |  |
| Yes | 0.80 | 0.41, 1.56 | 0.514 |  |  |  |

**Supplementary table 7. Factors associated with time to extubation in the whole population**

Univariable and multivariable Fine and Gray sub-distribution hazard models of the time to extubation with death as a competing risk. All univariable and multivariable models were adjusted on centers and performed on each of the imputed datasets. Estimates were pooled across imputations according to Rubin's rule. All independent variables with p<0.20 on univariable analyses were included in the multivariable model.

Abbreviations: 95% CI : 95% confidence interval ; ARDS :acute respiratory distress syndrome; BMI : body mass index; ECMO: extracorporeal membrane oxygenation; ICU: intensive care medicine; IMV: invasive mechanical ventilation; NMBA: neuromuscular blocking agent; P: p-value; PEEP : positive end-expiratory pressure; RRT: renal replacement therapy; SAPSII: simplified acute physiology score II; SHR : sub-distribution hazard ratio ; VAP: ventilator-associated pneumonia.

|  | **Univariable** | | | **Multivariable** | | |  |
| --- | --- | --- | --- | --- | --- | --- | --- |
| **Variable** | **SHR** | **95% CI** | **P** | **SHR** | **95% CI** | **P** |  |
| **Age (years)** | 0.98 | 0.96, 1 | **0.041** | 0.98 | 0.95, 1 | **0.035** |  |
| **Gender** |  |  |  |  |  |  |  |
| Woman | Ref | - | - |  |  |  |  |
| Man | 0.97 | 0.57, 1.67 | 0.917 |  |  |  |  |
| **BMI (kg/m²)** | 1.02 | 0.98, 1.06 | 0.399 |  |  |  |  |
| **MacCabe** |  |  |  |  |  |  |  |
| 1 | Ref | - | - |  |  |  |  |
| 2 | 0.89 | 0.52, 1.53 | 0.681 |  |  |  |  |
| 3 | 0.55 | 0.16, 1.9 | 0.345 |  |  |  |  |
| **SAPSII** | 0.98 | 0.97, 0.99 | **0.002** | 0.98 | 0.97, 1 | **0.017** |  |
| **Etiology** |  |  |  |  |  |  |  |
| Respiratory failure | Ref | - | - | Ref | - | - |  |
| Cardiac arrest | 2.11 | 0.87, 5.1 | 0.099 | 1.18 | 0.44, 3.11 | 0.7 |  |
| Other | 1.83 | 1.05, 3.2 | **0.033** | 1.13 | 0.59, 2.15 | 0.7 |  |
| **Sedatives** |  |  |  |  |  |  |  |
| Midazolam | Ref | - | - | Ref | - | - |  |
| Combination | 0.35 | 0.14, 0.86 | **0.021** | 0.27 | 0.1, 0.77 | **0.014** |  |
| Propofol | 2.36 | 1.04, 5.36 | **0.040** | 2.43 | 1.02, 5.82 | **0.046** |  |
| **Opioids** |  |  |  |  |  |  |  |
| Sufentanil | Ref | - | - |  |  |  |  |
| Other | 0.96 | 0.49, 1.86 | 0.896 |  |  |  |  |
| **PEEP (cmH2O)** | 0.98 | 0.91, 1.05 | 0.515 |  |  |  |  |
| **Plateau pressure (cmH2O)** | 0.97 | 0.93, 1.02 | 0.256 |  |  |  |  |
| **PaO₂/FiO₂** | 1.00 | 1, 1.01 | 0.412 |  |  |  |  |
| **TOF monitoring** |  |  |  |  |  |  |  |
| No | Ref | - | - |  |  |  |  |
| Yes | 1.16 | 0.43, 3.13 | 0.762 |  |  |  |  |
| **NMBA duration (days)** | 0.85 | 0.75, 0.96 | **0.008** | 0.78 | 0.7, 0.87 | **<0.001** |  |
| **NMBA dose (cis. eq.)** | 2.41 | 0.21, 28.31 | 0.484 |  |  |  |  |
| **Vasopressor** |  |  |  |  |  |  |  |
| No | Ref | - | - | Ref | - | - |  |
| Yes | 0.65 | 0.37, 1.12 | 0.117 | 0.61 | 0.34, 1.08 | 0.088 |  |
| **ECMO** |  |  |  |  |  |  |  |
| No | Ref | - | - | Ref | - | - |  |
| Yes | 0.37 | 0.12, 1.14 | 0.080 | 0.49 | 0.14, 1.76 | 0.3 |  |
| **RRT** |  |  |  |  |  |  |  |
| No | Ref | - | - |  |  |  |  |
| Yes | 1.08 | 0.29, 4.01 | 0.906 |  |  |  |  |
| **Prone position** | |  |  |  |  |  |  |
| No | | Ref | - | - |  |  |  |
| Yes | | 0.74 | 0.42, 1.28 | 0.279 |  |  |  |

**Supplementary table 8. Factors associated with time to extubation in the ARDS population.**

Univariable and multivariable Fine and Gray sub-distribution hazard models of the time to extubation with death as a competing risk. All univariable and multivariable models were adjusted on centers and performed on each of the imputed datasets. Estimates were pooled across imputations according to Rubin's rule. All independent variables with p<0.20 on univariable analyses were included in the multivariable model.

Abbreviations: 95% CI : 95% confidence interval ; ARDS :acute respiratory distress syndrome; BMI : body mass index; ECMO: extracorporeal membrane oxygenation; ICU: intensive care medicine; IMV: invasive mechanical ventilation; NMBA: neuromuscular blocking agent; P: p-value; PEEP : positive end-expiratory pressure; RRT: renal replacement therapy; SAPSII: simplified acute physiology score II; SHR : sub-distribution hazard ratio ; VAP: ventilator-associated pneumonia.

**Supplementary table 9 & 10. Factors associated with Ventilator-Associated Pneumonia**

|  | Univariable | | | Multivariable | | |
| --- | --- | --- | --- | --- | --- | --- |
| **Variable** | OR | 95% CI | P | OR | 95% CI | P |
| **Age (years)** | 0.99 | 0.97, 1 | 0.14 | 0.99 | 0.97, 1.02 | 0.6 |
| **Gender** |  |  |  |  |  |  |
| Woman | - | - |  |  |  |  |
| Man | 1.09 | 0.56, 2.09 | 0.8 |  |  |  |
| **BMI (kg/m²)** | 1.03 | 0.99, 1.08 | 0.2 |  |  |  |
| **MacCabe** |  |  |  |  |  |  |
| 1 | - | - |  |  |  |  |
| 2 | 0.74 | 0.37, 1.51 | 0.4 |  |  |  |
| 3 | 0.67 | 0.2, 2.25 | 0.5 |  |  |  |
| **Type of admission** |  |  |  |  |  |  |
| Planned surgery | - | - |  |  |  |  |
| Unplanned surgery | 3.00 | 0.32, 28.28 | 0.3 |  |  |  |
| Medical | 1.35 | 0.26, 6.9 | 0.7 |  |  |  |
| **SAPSII** | 0.98 | 0.97, 1 | **0.015** | 0.99 | 0.97, 1.01 | 0.3 |
| **Etiology** |  |  |  |  |  |  |
| Respiratory failure | - | - |  | - | - |  |
| Cardiac arrest | 0.59 | 0.25, 1.38 | 0.2 | 1.73 | 0.54, 5.55 | 0.4 |
| Other | 1.71 | 0.85, 3.45 | 0.13 | 3.03 | 1.21, 7.59 | **0.019** |
| **ARDS** |  |  |  |  |  |  |
| No | - | - |  |  |  |  |
| Yes | 2.27 | 1.2, 4.3 | **0.012** | 1.83 | 0.76, 4.44 | 0.2 |
| **Sedatives** |  |  |  |  |  |  |
| Midazolam | - | - |  | - | - |  |
| Combination | 1.97 | 0.84, 4.6 | 0.12 | 3.02 | 1.18, 7.76 | **0.022** |
| Propofol | 0.93 | 0.48, 1.83 | 0.8 | 1.38 | 0.65, 2.95 | 0.4 |
| **Opioids** |  |  |  |  |  |  |
| Sufentanil | - | - |  |  |  |  |
| Other | 0.57 | 0.26, 1.24 | 0.2 |  |  |  |
| **PEEP (cmH2O)** | 1.04 | 0.96, 1.12 | 0.3 |  |  |  |
| **Plateau pressure (cmH2O)** | 1.03 | 0.99, 1.08 | 0.2 |  |  |  |
| **PaO₂/FiO₂** | 1.00 | 0.99, 1 | 0.7 |  |  |  |
| **TOF monitoring** |  |  |  |  |  |  |
| No | - | - |  | - | - |  |
| Yes | 1.87 | 1.01, 3.44 | **0.046** | 1.33 | 0.61, 2.89 | 0.5 |
| **NMBA duration (days)** | 1.48 | 1.23, 1.77 | **<0.001** | 1.44 | 1.16, 1.79 | **0.001** |
| **NMBA dose (cis. eq.)** | 3.63 | 0.26, 50 | 0.3 |  |  |  |
| **Vasopressor** |  |  |  |  |  |  |
| No | - | - |  |  |  |  |
| Yes | 0.62 | 0.3, 1.28 | 0.2 |  |  |  |
| **ECMO** |  |  |  |  |  |  |
| No | - | - |  |  |  |  |
| Yes | 1.05 | 0.29, 3.77 | >0.9 |  |  |  |
| **RRT** |  |  |  |  |  |  |
| No | - | - |  |  |  |  |
| Yes | 1.04 | 0.31, 3.52 | >0.9 |  |  |  |

**Supplementary table 9. Factors associated with Ventilator-associated pneumonia in the whole population**

Univariable and multivariable logistic regression models were fitted to compute the odds ratio (OR) for the occurrence of ventilator-associated pneumonia. All univariable and multivariable models were clustered on centers and performed on each of the imputed datasets. Estimates were pooled across imputations according to the Rubin’s rule with robust confidence intervals (95% CI) with cluster-adjusted variance. All independent variables with p<0.20 on univariable analyses were included in the multivariable model.

Abbreviations. ARDS: acute respiratory distress syndrome; BMI: body mass index; ECMO: extracorporeal membrane oxygenation; ICU: intensive care medicine; IMV: invasive mechanical ventilation; NMBA: neuromuscular blocking agent; PEEP: positive end-expiratory pressure; RRT: renal replacement therapy; SAPSII: simplified acute physiology score II; VAP: ventilator-associated pneumonia.

|  | **Univariable** | | | **Multivariable** | | |  |
| --- | --- | --- | --- | --- | --- | --- | --- |
| **Variable** | **OR** | **95% CI** | **P** | **OR** | **95% CI** | **P** |  |
| **Age (years)** | 0.99 | 0.96, 1.01 | **0.3** | 0.98 | 0.95, 1 | **0.035** |  |
| **Gender** |  |  |  |  |  |  |  |
| Woman | Ref | - | - |  |  |  |  |
| Man | 0.96 | 0.41, 2.24 | >0.9 |  |  |  |  |
| **BMI (kg/m²)** | 1.03 | 0.97, 1.1 | 0.3 |  |  |  |  |
| **MacCabe** |  |  |  |  |  |  |  |
| 1 | Ref | - | - |  |  |  |  |
| 2 | 0.65 | 0.28, 1.54 | 0.3 |  |  |  |  |
| 3 | 0.49 | 0.13, 1.84 | 0.3 |  |  |  |  |
| **SAPSII** | 0.99 | 0.97, 1.01 | 0.3 |  |  |  |  |
| **Etiology** |  |  |  |  |  |  |  |
| Respiratory failure | Ref | - | - | Ref | - | - |  |
| Cardiac arrest | 0.99 | 0.18, 5.47 | >0.9 | 1.24 | 0.19, 8.12 | 0.8 |  |
| Other | 3.82 | 1.56, 9.38 | **0.004** | 4.29 | 1.62, 11.39 | **0.004** |  |
| **Sedatives** |  |  |  |  |  |  |  |
| Midazolam | Ref | - | - |  |  |  |  |
| Combination | 1.50 | 0.47, 4.76 | **0.5** |  |  |  |  |
| Propofol | 0.91 | 0.37, 2.23 | **0.8** |  |  |  |  |
| **Opioids** |  |  |  |  |  |  |  |
| Sufentanil | Ref | - | - |  |  |  |  |
| Other | 0.72 | 0.26, 2.01 | 0.5 |  |  |  |  |
| **PEEP (cmH2O)** | 1.00 | 0.89, 1.11 | >0.9 |  |  |  |  |
| **Plateau pressure (cmH2O)** | 1.01 | 0.94, 1.09 | 0.8 |  |  |  |  |
| **PaO₂/FiO₂** | 1.00 | 0.99, 1.01 | 0.8 |  |  |  |  |
| **TOF monitoring** |  |  |  |  |  |  |  |
| No | Ref | - | - |  |  |  |  |
| Yes | 1.25 | 0.59, 2.62 | 0.6 |  |  |  |  |
| **NMBA duration (days)** | 1.31 | 1.1, 1.57 | **0.003** | 1.34 | 1.07, 1.67 | **0.011** |  |
| **NMBA dose (cis. eq.)** | 4.84 | 0.23, 100.58 | 0.3 |  |  |  |  |
| **Vasopressor** |  |  |  |  |  |  |  |
| No | Ref | - | - |  |  |  |  |
| Yes | 0.70 | 0.29, 1.69 | 0.4 |  |  |  |  |
| **ECMO** |  |  |  |  |  |  |  |
| No | Ref | - | - |  |  |  |  |
| Yes | 1.64 | 0.34, 7.98 | 0.5 |  |  |  |  |
| **RRT** |  |  |  |  |  |  |  |
| No | Ref | - | - |  |  |  |  |
| Yes | 1.07 | 0.29, 3.88 | >0.9 |  |  |  |  |
| **Prone position** | |  |  |  |  |  |  |
| No | | Ref | - | - |  |  |  |
| Yes | | 1.77 | 0.84, 3.73 | 0.13 | 1.00 | 0.39, 2.54 | >0.9 |

**Supplementary table 10. Factors associated with Ventilator-associated pneumonia in ARDS patients**

Univariable and multivariable logistic regression models were fitted to compute the odds ratio (OR) for the occurrence of ventilator-associated pneumonia. All univariable and multivariable models were clustered on centers and performed on each of the imputed datasets. Estimates were pooled across imputations according to the Rubin’s rule with robust confidence intervals (95% CI) with cluster-adjusted variance. All independent variables with p<0.20 on univariable analyses were included in the multivariable model.

Abbreviations. ARDS: acute respiratory distress syndrome; BMI: body mass index; ECMO: extracorporeal membrane oxygenation; ICU: intensive care medicine; IMV: invasive mechanical ventilation; NMBA: neuromuscular blocking agent; PEEP: positive end-expiratory pressure; RRT: renal replacement therapy; SAPSII: simplified acute physiology score II; VAP: ventilator-associated pneumonia.

**Supplementary table 11 & 12. Factors associated with pressure ulcers**

|  | **Univariable** | | | **Multivariable** | | |
| --- | --- | --- | --- | --- | --- | --- |
| **Variable** | **OR** | **95% CI** | **P** | **OR** | **95% CI** | **P** |
| **Age (years)** | 1.01 | 0.99, 1.04 | 0.2 |  |  |  |
| **Gender** |  |  |  |  |  |  |
| Woman | - | - |  |  |  |  |
| Man | 0.97 | 0.44, 2.16 | >0.9 |  |  |  |
| **BMI (kg/m²)** | 0.97 | 0.91, 1.04 | 0.4 |  |  |  |
| **MacCabe** |  |  |  |  |  |  |
| 1 | - | - |  |  |  |  |
| 2 | 0.49 | 0.17, 1.44 | 0.2 |  |  |  |
| 3 | 0.49 | 0.1, 2.38 | 0.4 |  |  |  |
| **Type of admission** |  |  |  |  |  |  |
| Planned surgery | - | - |  |  |  |  |
| Unplanned surgery | 0.50 | 0.03, 7.24 | 0.6 |  |  |  |
| Medical | 0.74 | 0.14, 4.01 | 0.7 |  |  |  |
| **SAPSII** | 0.98 | 0.97, 1 | 0.054 | 0.99 | 0.97, 1.01 | 0.5 |
| **Etiology** |  |  |  |  |  |  |
| Respiratory failure | - | - |  | - | - |  |
| Cardiac arrest | 0.45 | 0.15, 1.32 | 0.14 | 1.31 | 0.34, 5.02 | 0.7 |
| Other | 1.08 | 0.45, 2.6 | 0.9 | 1.62 | 0.53, 4.9 | 0.4 |
| **ARDS** |  |  |  |  |  |  |
| No | - | - |  |  |  |  |
| Yes | 4.14 | 1.52, 11.25 | **0.006** | 3.81 | 1.08, 13.41 | **0.038** |
| **Sedatives** |  |  |  |  |  |  |
| Midazolam | - | - |  |  |  |  |
| Combination | 0.80 | 0.25, 2.57 | 0.7 |  |  |  |
| Propofol | 0.57 | 0.23, 1.41 | 0.2 |  |  |  |
| **Opioids** |  |  |  |  |  |  |
| Sufentanil | - | - |  |  |  |  |
| Other | 1.29 | 0.54, 3.09 | 0.6 |  |  |  |
| **PEEP (cmH2O)** | 1.09 | 1, 1.19 | 0.061 | 1.03 | 0.93, 1.14 | 0.6 |
| **Plateau pressure (cmH2O)** | 0.96 | 0.91, 1.02 | 0.2 |  |  |  |
| **PaO₂/FiO₂** | 1.00 | 0.99, 1 | 0.3 |  |  |  |
| **TOF monitoring** |  |  |  |  |  |  |
| No | - | - |  |  |  |  |
| Yes | 2.52 | 1.13, 5.6 | **0.024** | 1.72 | 0.69, 4.3 | 0.2 |
| **NMBA duration (days)** | 1.38 | 1.19, 1.6 | **<0.001** | 1.22 | 1.04, 1.44 | **0.018** |
| **NMBA dose (cis. eq.)** | 6.79 | 0.33, 139.62 | 0.2 |  |  |  |
| **Vasopressor** |  |  |  |  |  |  |
| No | - | - |  |  |  |  |
| Yes | 1.28 | 0.49, 3.33 | 0.6 |  |  |  |
| **ECMO** |  |  |  |  |  |  |
| No | - | - |  | - | - |  |
| Yes | 4.98 | 1.32, 18.81 | **0.020** | 6.62 | 1.55, 28.25 | **0.012** |
| **RRT** |  |  |  |  |  |  |
| No | - | - |  |  |  |  |
| Yes | 1.09 | 0.29, 4.03 | 0.9 |  |  |  |

**Supplementary table 11. Factors associated with pressure ulcers in the whole population**

Univariable and multivariable logistic regression models were fitted to compute the odds ratio (OR) for the occurrence of pressure ulcers. All univariable and multivariable models were clustered on centers and performed on each of the imputed datasets. Estimates were pooled across imputations according to the Rubin’s rule with robust confidence intervals (95% CI) with cluster-adjusted variance. All independent variables with p<0.20 on univariable analyses were included in the multivariable model.

Abbreviations. ARDS: acute respiratory distress syndrome; BMI: body mass index; ECMO: extracorporeal membrane oxygenation; ICU: intensive care medicine; IMV: invasive mechanical ventilation; NMBA: neuromuscular blocking agent; PEEP: positive end-expiratory pressure; RRT: renal replacement therapy; SAPSII: simplified acute physiology score II.

|  | **Univariable** | | | **Multivariable** | | |  |
| --- | --- | --- | --- | --- | --- | --- | --- |
| **Variable** | **OR** | **95% CI** | **P** | **OR** | **95% CI** | **P** |  |
| **Age (years)** | 1.01 | 0.98, 1.04 | 0.5 |  |  |  |  |
| **Gender** |  |  |  |  |  |  |  |
| Woman | Ref | - | - |  |  |  |  |
| Man | 0.65 | 0.26, 1.63 | 0.4 |  |  |  |  |
| **BMI (kg/m²)** | 0.98 | 0.91, 1.05 | 0.5 |  |  |  |  |
| **MacCabe** |  |  |  |  |  |  |  |
| 1 | Ref | - | - | Ref | - | - |  |
| 2 | 0.65 | 0.26, 1.63 | 0.4 | 0.65 | 0.26, 1.63 | 0.4 |  |
| 3 | 0.98 | 0.91, 1.05 | 0.5 | 0.98 | 0.91, 1.05 | 0.5 |  |
| **SAPSII** | 0.99 | 0.97, 1.01 | 0.5 |  |  |  |  |
| **Etiology** |  |  |  |  |  |  |  |
| Respiratory failure | Ref | - | - |  |  |  |  |
| Cardiac arrest | 0.50 | 0.06, 4.43 | 0.5 |  |  |  |  |
| Other | 1.65 | 0.59, 4.66 | 0.3 |  |  |  |  |
| **Sedatives** |  |  |  |  |  |  |  |
| Midazolam | Ref | - | - |  |  |  |  |
| Combination | 0.91 | 0.24, 3.46 | 0.9 |  |  |  |  |
| Propofol | 0.66 | 0.23, 1.88 | 0.4 |  |  |  |  |
| **Opioids** |  |  |  |  |  |  |  |
| Sufentanil | Ref | - | - |  |  |  |  |
| Other | 0.93 | 0.33, 2.62 | 0.9 |  |  |  |  |
| **PEEP (cmH2O)** | 1.05 | 0.93, 1.19 | 0.4 |  |  |  |  |
| **Plateau pressure (cmH2O)** | 0.95 | 0.87, 1.04 | 0.3 |  |  |  |  |
| **PaO₂/FiO₂** | 1.00 | 0.99, 1.01 | >0.9 |  |  |  |  |
| **TOF monitoring** |  |  |  |  |  |  |  |
| No | Ref | - | - | Ref | - | - |  |
| Yes | 2.86 | 1.11, 7.39 | **0.030** | 2.81 | 0.89, 8.86 | 0.078 |  |
| **NMBA duration (days)** | 1.29 | 1.1, 1.52 | **0.002** | 1.13 | 0.94, 1.37 | 0.2 |  |
| **NMBA dose (cis. eq.)** | 3.41 | 0.08, 137.59 | 0.5 |  |  |  |  |
| **Vasopressor** |  |  |  |  |  |  |  |
| No | Ref | - | - |  |  |  |  |
| Yes | 1.54 | 0.53, 4.54 | 0.4 |  |  |  |  |
| **ECMO** |  |  |  |  |  |  |  |
| No | Ref | - | - |  |  |  |  |
| Yes | 8.52 | 1.46, 49.75 | **0.018** | 10.77 | 1.09, 106.42 | **0.042** |  |
| **RRT** |  |  |  |  |  |  |  |
| No | Ref | - | - |  |  |  |  |
| Yes | 1.42 | 0.28, 7.27 | 0.7 |  |  |  |  |
| **Prone position** | |  |  |  |  |  |  |
| No | | Ref | - | - |  |  |  |
| Yes | | 3.65 | 1.33, 10.02 | **0.013** | 2.37 | 0.72, 7.85 | 0.2 |

**Supplementary table 12. Factors associated with pressure ulcers in ARDS patients**

Univariable and multivariable logistic regression models were fitted to compute the odds ratio (OR) for the occurrence of pressure ulcers. All univariable and multivariable models were clustered on centers and performed on each of the imputed datasets. Estimates were pooled across imputations according to the Rubin’s rule with robust confidence intervals (95% CI) with cluster-adjusted variance. All independent variables with p<0.20 on univariable analyses were included in the multivariable model.

Abbreviations. ARDS: acute respiratory distress syndrome; BMI: body mass index; ECMO: extracorporeal membrane oxygenation; ICU: intensive care medicine; IMV: invasive mechanical ventilation; NMBA: neuromuscular blocking agent; PEEP: positive end-expiratory pressure; RRT: renal replacement therapy; SAPSII: simplified acute physiology score II.

**Supplementary table 13 & 14. Factors associated with ICU-acquired weakness**

|  | **Univariable** | | | **Multivariable** | | |
| --- | --- | --- | --- | --- | --- | --- |
| **Variable** | **OR** | **95% CI** | **P** | **OR** | **95% CI** | **P** |
| **Age (years)** | 1.00 | 0.98, 1.02 | 0.7 |  |  |  |
| **Gender** |  |  |  |  |  |  |
| Woman | - | - |  |  |  |  |
| Man | 0.95 | 0.5, 1.84 | 0.9 |  |  |  |
| **BMI (kg/m²)** | 1.04 | 0.99, 1.09 | 0.10 | 1.05 | 1, 1.11 | 0.061 |
| **MacCabe** |  |  |  |  |  |  |
| 1 | - | - |  |  |  |  |
| 2 | 0.79 | 0.38, 1.66 | 0.5 |  |  |  |
| 3 | 1.46 | 0.53, 3.98 | 0.5 |  |  |  |
| **Type of admission** |  |  |  |  |  |  |
| Planned surgery | - | - |  | - | - |  |
| Unplanned surgery | 0.64 | 0.08, 4.93 | 0.7 | 0.42 | 0.03, 7.22 | 0.5 |
| Medical | 0.37 | 0.1, 1.33 | 0.13 | 0.50 | 0.11, 2.21 | 0.4 |
| **SAPSII** | 0.99 | 0.97, 1 | 0.14 | 1.00 | 0.98, 1.02 | >0.9 |
| **Etiology** |  |  |  |  |  |  |
| Respiratory failure | - | - |  |  |  |  |
| Cardiac arrest | 0.54 | 0.22, 1.32 | 0.2 | 1.30 | 0.41, 4.08 | 0.7 |
| Other | 1.91 | 0.95, 3.84 | 0.069 | 3.35 | 1.28, 8.74 | **0.014** |
| **ARDS** |  |  |  |  |  |  |
| No | - | - |  | - | - |  |
| Yes | 3.14 | 1.56, 6.32 | **0.002** | 3.10 | 1.29, 7.49 | **0.012** |
| **Sedatives** |  |  |  |  |  |  |
| Midazolam | - | - |  | - | - |  |
| Combination | 0.76 | 0.31, 1.87 | 0.6 | 1.23 | 0.46, 3.3 | 0.7 |
| Propofol | 0.44 | 0.21, 0.91 | **0.027** | 0.64 | 0.26, 1.53 | 0.3 |
| **Opioids** |  |  |  |  |  |  |
| Sufentanil | - | - |  | - | - |  |
| Other | 0.38 | 0.15, 0.96 | **0.041** | 0.46 | 0.16, 1.35 | 0.2 |
| **PEEP (cmH2O)** | 1.06 | 0.98, 1.14 | 0.2 |  |  |  |
| **Plateau pressure (cmH2O)** | 1.00 | 0.96, 1.05 | >0.9 |  |  |  |
| **PaO₂/FiO₂** | 1.00 | 0.99, 1 | 0.2 |  |  |  |
| **TOF monitoring** |  |  |  |  |  |  |
| No | - | - |  | - | - |  |
| Yes | 3.35 | 1.77, 6.34 | **<0.001** | 2.90 | 1.2, 7.01 | **0.018** |
| **NMBA duration (days)** | 1.34 | 1.12, 1.61 | **0.001** | 1.18 | 0.97, 1.44 | 0.092 |
| **NMBA dose (cis. eq.)** | 2.46 | 0.18, 34.03 | 0.5 |  |  |  |
| **Vasopressor** |  |  |  |  |  |  |
| No | - | - |  |  |  |  |
| Yes | 1.79 | 0.74, 4.35 | 0.2 |  |  |  |
| **ECMO** |  |  |  |  |  |  |
| No | - | - |  | - | - |  |
| Yes | 4.20 | 1.42, 12.43 | **0.010** | 5.68 | 1.64, 19.7 | **0.007** |
| **RRT** |  |  |  |  |  |  |
| No | - | - |  | - | - |  |
| Yes | 2.65 | 1.01, 6.96 | **0.048** | 2.12 | 0.69, 6.52 | 0.2 |

**Supplementary table 13. Factors associated with ICU-acquired weakness in the whole population**

Univariable and multivariable logistic regression models were fitted to compute the odds ratio (OR) for the occurrence of ICU-acquired weakness. All univariable and multivariable models were clustered on centers and performed on each of the imputed datasets. Estimates were pooled across imputations according to the Rubin’s rule with robust confidence intervals (95% CI) with cluster-adjusted variance. All independent variables with p<0.20 on univariable analyses were included in the multivariable model.

Abbreviations: ARDS: acute respiratory distress syndrome; BMI: body mass index; ECMO: extracorporeal membrane oxygenation; ICU: intensive care medicine; IMV: invasive mechanical ventilation; NMBA: neuromuscular blocking agent; PEEP: positive end-expiratory pressure; RRT: renal replacement therapy; SAPSII: simplified acute physiology score II.

|  | **Univariable** | | | **Multivariable** | | |  |
| --- | --- | --- | --- | --- | --- | --- | --- |
| **Variable** | **OR** | **95% CI** | **P** | **OR** | **95% CI** | **P** |  |
| **Age (years)** | 0.98 | 0.96, 1.01 | 0.2 |  |  |  |  |
| **Gender** |  |  |  |  |  |  |  |
| Woman | Ref | - | - |  |  |  |  |
| Man | 0.78 | 0.33, 1.81 | 0.6 |  |  |  |  |
| **BMI (kg/m²)** | 1.04 | 0.98, 1.11 | 0.2 |  |  |  |  |
| **MacCabe** |  |  |  |  |  |  |  |
| 1 | Ref | - | - |  |  |  |  |
| 2 | 0.62 | 0.26, 1.47 | 0.3 |  |  |  |  |
| 3 | 0.99 | 0.3, 3.29 | >0.9 |  |  |  |  |
| **SAPSII** | 0.99 | 0.98, 1.01 | 0.5 |  |  |  |  |
| **Etiology** |  |  |  |  |  |  |  |
| Respiratory failure | Ref | - | - | Ref | - | - |  |
| Cardiac arrest | 0.85 | 0.16, 4.67 | 0.9 | 0.78 | 0.2, 3.09 | 0.7 |  |
| Other | 3.19 | 1.2, 8.46 | **0.020** | 3.23 | 1, 10.39 | **0.050** |  |
| **Sedatives** |  |  |  |  |  |  |  |
| Midazolam | Ref | - | - |  |  |  |  |
| Combination | 1.29 | 0.43, 3.89 | 0.6 |  |  |  |  |
| Propofol | 0.63 | 0.26, 1.52 | 0.3 |  |  |  |  |
| **Opioids** |  |  |  |  |  |  |  |
| Sufentanil | Ref | - | - | Ref | - | - |  |
| Other | 0.47 | 0.17, 1.3 | 0.14 | 0.61 | 0.2, 1.83 | 0.4 |  |
| **PEEP (cmH2O)** | 0.99 | 0.9, 1.09 | 0.8 |  |  |  |  |
| **Plateau pressure (cmH2O)** | 0.98 | 0.92, 1.04 | 0.5 |  |  |  |  |
| **PaO₂/FiO₂** | 1.00 | 0.99, 1.01 | 0.7 |  |  |  |  |
| **TOF monitoring** |  |  |  |  |  |  |  |
| No | Ref | - | - |  |  |  |  |
| Yes | 1.72 | 0.79, 3.73 | 0.2 |  |  |  |  |
| **NMBA duration (days)** | 1.26 | 1.03, 1.53 | **0.022** | 1.17 | 0.93, 1.47 | 0.2 |  |
| **NMBA dose (cis. eq.)** | 1.30 | 0.05, 31.35 | 0.9 |  |  |  |  |
| **Vasopressor** |  |  |  |  |  |  |  |
| No | Ref | - | - |  |  |  |  |
| Yes | 1.92 | 0.74, 4.99 | 0.2 |  |  |  |  |
| **ECMO** |  |  |  |  |  |  |  |
| No | Ref | - | - | Ref | - | - |  |
| Yes | 11.41 | 1.19, 108.97 | **0.035** | 15.44 | 1.16, 204.93 | **0.038** |  |
| **RRT** |  |  |  |  |  |  |  |
| No | Ref | - | - | Ref | - | - |  |
| Yes | 4.12 | 1.13, 14.98 | **0.032** | 3.22 | 0.7, 14.75 | 0.13 |  |
| **Prone position** | |  |  |  |  |  |  |
| No | | Ref | - | - | Ref | - | - |
| Yes | | 3.40 | 1.54, 7.49 | **0.003** | 2.59 | 0.91, 7.4 | 0.074 |

**Supplementary table 14. Factors associated with ICU-acquired weakness in the ARDS patients**

Univariable and multivariable logistic regression models were fitted to compute the odds ratio (OR) for the occurrence of ICU-acquired weakness. All univariable and multivariable models were clustered on centers and performed on each of the imputed datasets. Estimates were pooled across imputations according to the Rubin’s rule with robust confidence intervals (95% CI) with cluster-adjusted variance. All independent variables with p<0.20 on univariable analyses were included in the multivariable model.

Abbreviations: ARDS: acute respiratory distress syndrome; BMI: body mass index; ECMO: extracorporeal membrane oxygenation; ICU: intensive care medicine; IMV: invasive mechanical ventilation; NMBA: neuromuscular blocking agent; PEEP: positive end-expiratory pressure; RRT: renal replacement therapy; SAPSII: simplified acute physiology score II.
